# Long non-coding RNA urothelial carcinoma associated 1 (UCA1) mediates radiation response in prostate cancer

**DOI:** 10.18632/oncotarget.13576

**Published:** 2016-11-24

**Authors:** Alireza Fotouhi Ghiam, Samira Taeb, Xiaoyong Huang, Vincent Huang, Jessica Ray, Seville Scarcello, Christianne Hoey, Sahar Jahangiri, Emmanouil Fokas, Andrew Loblaw, Robert G. Bristow, Danny Vesprini, Paul Boutros, Stanley K. Liu

**Affiliations:** ^1^ Sunnybrook Research Institute, Sunnybrook Health Sciences Centre, Toronto, Canada; ^2^ Department of Radiation Oncology, University of Toronto, Canada; ^3^ Ontario Institute for Cancer Research, University of Toronto, Canada; ^4^ Department of Medical Biophysics, University of Toronto, Canada; ^5^ Oxford Institute for Radiation Oncology, University of Oxford, UK

**Keywords:** lncRNA, UCA1, irradiation resistant, prostate cancer, biomarker

## Abstract

Radioresistance remains a significant obstacle in the treatment of Prostate Cancer (PCa). To simulate the clinical scenario of irradiation resistance (IRR), we created DU145-IRR PCa cell lines by treatment with 2 Gy daily IR for 59 fractions. DU145-IRR cells acquired an aggressive phenotype as evidenced by increased clonogenic survival, tumorigenic potential and invasiveness. We performed transcriptome profiling to discover dysregulated genes in DU145-IRR cells and identified the long non-coding RNA (lncRNA), Urothelial carcinoma-associated 1 (UCA1). We first investigated the role of UCA1 in radiation response and found that UCA1 abundance was significantly higher in DU145-IRR cells compared to control cells. UCA1 siRNA-knockdown reversed the aggressive phenotype and significantly increased sensitivity to IR. UCA1 depletion inhibited growth, induced cell cycle arrest at the G2/M transition and decreased activation of the pro-survival Akt pathway. We then studied the clinical significance of UCA1 expression in two independent cohorts of PCa patients: MSKCC (130 patients) and CPC-GENE (209 patients). UCA1 over-expression was associated with decreased 5-year disease-free survival in MSKCC patients (HR = 2.9; p = 0.007) and a trend toward lower biochemical recurrence-free survival in CPC-GENE patients (HR = 2.7; p = 0.05). We showed for the first time that UCA1 depletion induces radiosensitivity, decreases proliferative capacity and disrupts cell cycle progression, which may occur through altered Akt signaling and induced cell cycle arrest at the G2/M transition. Our results indicate that UCA1 might have prognostic value in PCa and be a potential therapeutic target.

## INTRODUCTION

Prostate cancer (PCa) is the most common non-dermatological cancer and the second-leading cause of cancer-related death in men [1]. Radiotherapy (RT) including external beam radiotherapy (EBRT) and brachytherapy is one of the standard treatment modalities for PCa. Despite significant advances in RT techniques, a significant portion of PCa patients can still fail RT due to the intrinsic radioresistance of a subpopulation of cells within the tumor [2]. The therapeutic resistance of prostate tumors is complex and involves a multifactorial process [2] that is confounded by the genomic diversity of the disease [3, 4]. There is a clear need to better understand the mechanisms that impact radiosensitivity of this tumor type to improve treatment outcome and minimize RT-induced toxicity.

Long non-coding RNAs (lncRNAs) are a group of non-coding transcripts of more than 200 nucleotides that function in a broad range of cellular processes, such as cell growth, survival, migration, invasion, and differentiation [5]. They are also involved in tumorigenesis and metastasis as well as radiotherapy and chemotherapy resistance. [6–8]. LncRNAs are highly deregulated in tumors and have been found to act as tumor suppressors or oncogenes [7]. Recent studies indicate that lncRNAs play important biological and clinical roles in PCa; and overexpression of oncogenic lncRNAs may promote tumor cell proliferation and metastasis [9, 10].

Urothelial carcinoma associated 1 (UCA1) is a recently identified lncRNA with aberrant expression in various types of cancer [11]. UCA1 expression regulates cancer cell proliferation, migration, and invasion [11]. Recent studies have shown that UCA1 is a functional oncogenic lncRNA upregulated in several malignancies [11]. UCA1 abundance is correlated with clinico-pathological parameters (particularly in bladder cancer), suggesting higher abundance as a marker of higher histologic grade [11, 12].

The biological role and clinical significance of UCA1 in PCa carcinogenesis and therapy response are not yet well understood. Here, for the first time, we show that irradiation-resistant (IRR) PCa cells express a significantly higher level of UCA1 *in vitro* and UCA1-knockdown reverses the aggressive phenotype and improves radiosensitivity. Our data suggest that UCA1 contributes to radiotherapy resistance through regulation of the PI3K/Akt pathway. The biological role of UCA1 in tumor proliferation, invasion, tumorigenesis, cell cycle progression and DNA repair were investigated. We showed that the higher expression of UCA1 is associated with unfavorable outcome in two separate cohorts of PCa patients.

## RESULTS

### Irradiation-resistant DU145 cancer cells possess an aggressive phenotype

**Figure 1:**
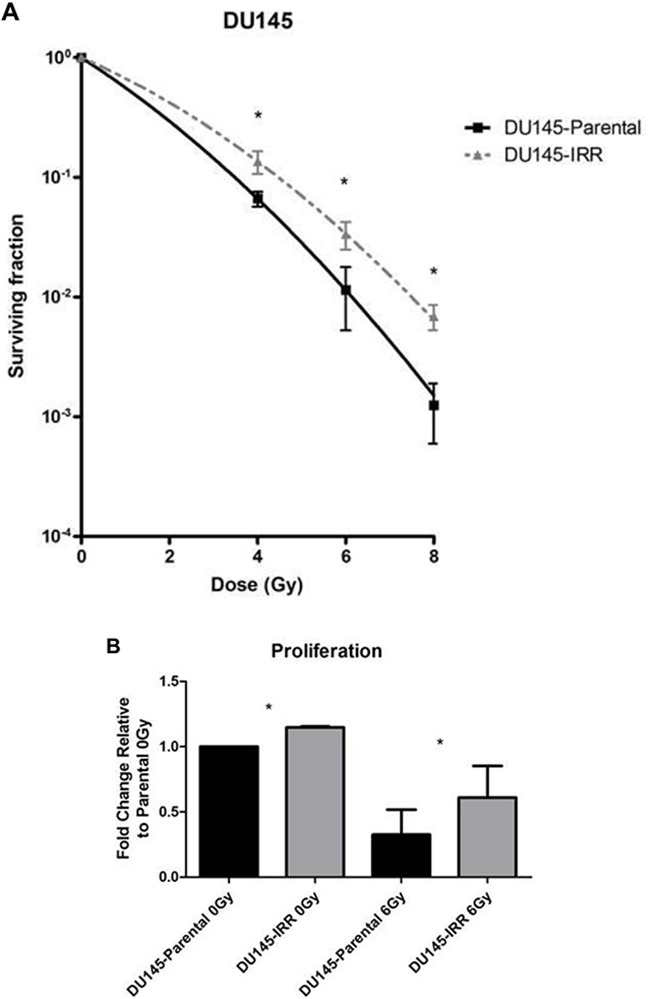

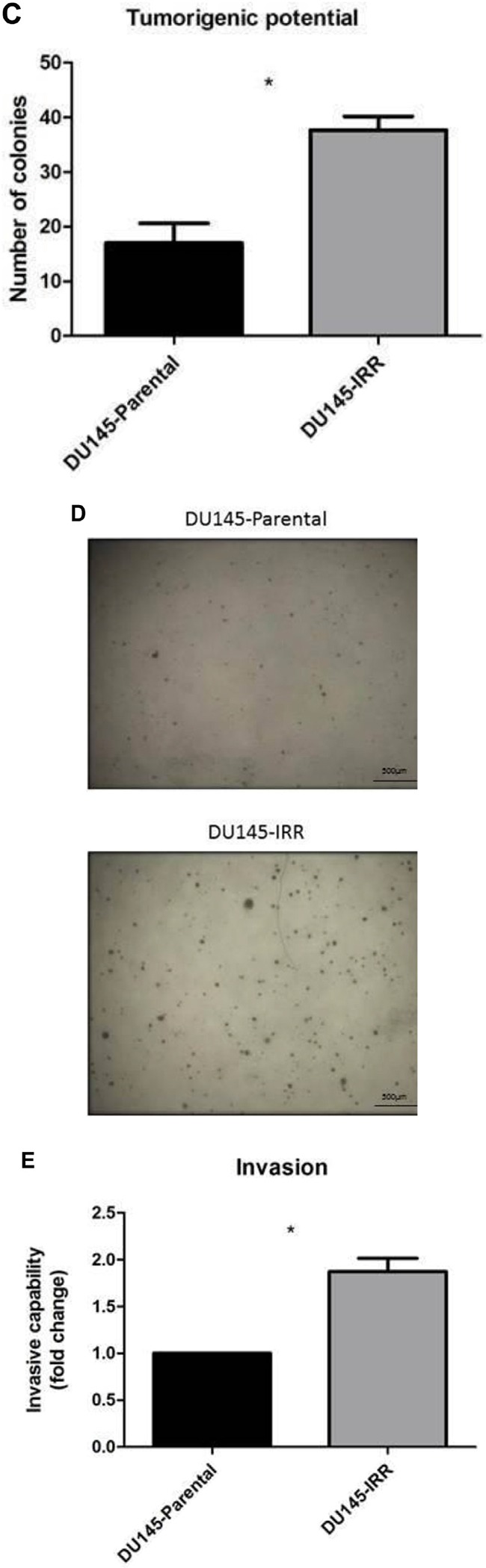

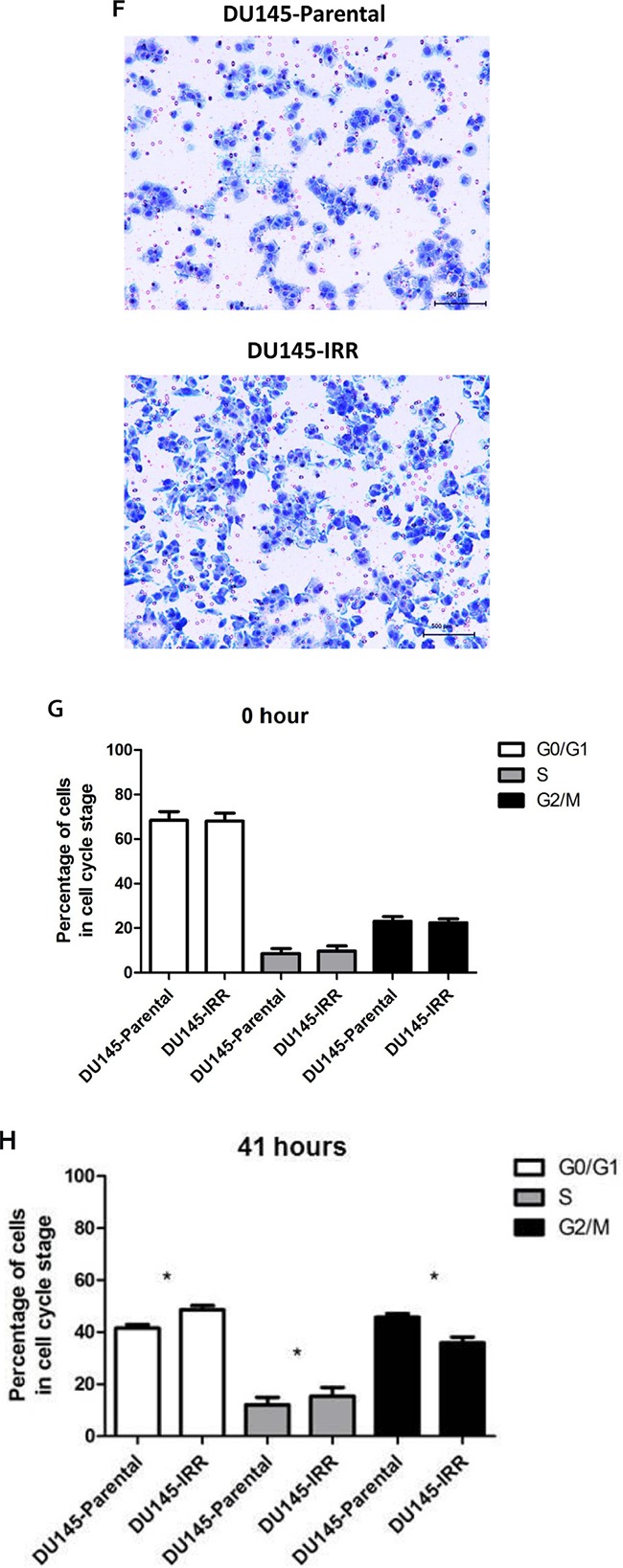
DU145 cells surviving RT are IRR and have an aggressive phenotype characterized by increased proliferation, invasive potential, and impaired G2-M cell cycle arrest **A**. DU145 cells were mock irradiated with 0 Gy (DU145-Parental) or irradiated with a total of 2 Gy × 59 daily fractions of IR (DU145-IRR), radiation clonogenic survival assays were performed, and surviving fraction fitted to the linear-quadratic equation. **B**. Cell counts of viable DU145-Parental and DU145-IRR cells following mock IR (0 Gy) or 6 Gy dose of IR. **C**. Soft agar assay of DU145-Parental and DU145-IRR cells. **D**. A representative soft agar experiment out of three experiments (scale bar denotes 500μm). **E**. Matrigel transwell invasion assay of DU145-Parental and DU145-IRR cells. **F**. A representative invasion assay out of three experiments (scale bar denotes 500μm). Cell-cycle profiles of DU145-Parental and DU145-IRR cells following a 6 Gy dose of IR at **G**. 0 hour or **H**. 41 hours. Means, SDs, and differences of statistical significance are denoted by * (p < 0.05); n = 3 independent experiments for each experiment.

To simulate the clinical scenario of resistance to conventional fractionated RT, DU145 PCa cells were mock irradiated with 0 Gy (DU145-Parental) or irradiated with 2 Gy daily fractions of IR over several weeks (DU145-IRR). Surviving cells were pooled and radiation clonogenic survival curves revealed that DU145-IRR cells were significantly more resistant to an acute exposure to 4 Gy, 6 Gy and 8 Gy of IR compared to DU145-Parental control cells (ANOVA and t-test; p values < 0.05; Figure 1A). Visual differences were apparent between the samples, with the cells in DU145-IRR appearing larger and more dense (Supplementary Figure S1). We further characterized the other phenotypic characteristics of DU145-IRR cells in regards to proliferation, soft agar colony formation, invasiveness and cell cycle profiles.

Proliferation is an important contributor to cancer development and progression. DU145-IRR cells proliferated more quickly compared with DU145-Parental cells before [fold change relative to parental 0 Gy: 1.23 ± 0.09-fold (IRR) *vs*. 1.0-fold (parental); p = 0.02; Figure 1B] and following 6Gy IR [fold change relative to parental 0 Gy: 0.61 ± 0.28-fold (IRR) *vs*. 0.32 ± 0.22-fold (parental); p = 0.01; Figure 1B].

We then used the soft agar colony formation assay to evaluate anchorage-independent cellular growth *in vitro*. The soft agar assay is the gold standard for cellular transformation and growth in soft agar is strongly correlated to tumorigenicity in animals [13]. Consistent with a more aggressive phenotype, tumorigenic potential was significantly enhanced in DU145-IRR cells compared with DU145-Parental cells (mean number of colonies 37.7 ± 1.4 (IRR) *vs*. 17 ± 2.1 (Parental); p = 0.001; Figure 1C, 1D).

Invasiveness is an important property for an aggressive phenotype in cancer cells, which increases the propensity for regional lymphatic and distant metastatic spread, and may be enriched in radiation-resistant cancers [14]. The Matrigel transwell invasion assay revealed that DU145-IRR cells invaded more readily than DU145-Parental cells (1.87 ± 0.1-fold (IRR) *vs*. 1.0-fold (Parental); p = 0.008; Figure 1E, 1F).

Flow cytometric analysis revealed no significant differences between the DU145-Parental and DU145-IRR cell cycle profiles before IR [G0/G1 phase: 68 ± 5.1% (IRR) *vs*. 68.3 ± 5.6% (Parental); S phase: 9.6 ± 3.3% (IRR) *vs*. 8.6 ± 3.1% (Parental); G2/M phase: 22.34 ± 2.5% (IRR) *vs*. 23 ± 3.1% (Parental); p values = ns; Figure 1G]. However, 41 hours following a 10 Gy dose of IR, a significantly greater percentage of DU145-IRR cells remained in the G0/G1 [48.6 ± 2.2% (IRR) *vs*. 41.5 ± 1.9% (parental); p = 0.009] and S phases [15.4 ± 4.8% (IRR) *vs*. 12.1 ± 3.9% (parental); p = 0.03] compared with DU145-Parental cells, and correspondingly, a fewer percentage of DU145-IRR cells were present in the G2/M phase [35.8 ± 3.2% (IRR) *vs*. 45.7 ± 2.1% (parental); p = 0.02; Figure 1H]. Collectively, these results indicate that the DU145-IRR cells have acquired a more aggressive phenotype consisting of increased proliferation and tumorigenicity, enhanced invasive potential and impaired G2/M cell-cycle arrest.

### Identification of differentially expressed genes (DEGs) related to radioresistance

Gene array analysis showed that 62 genes were up-regulated and 73 genes were down-regulated by ≥ 2-fold in DU145-IRR cells compared to DU145-Parental cells. The top 10 up and top 10 down regulated genes are shown in Table 1. This list includes genes involved in signal transduction, cell cycle regulation, metabolic enzyme pathway, and cell adhesion/motility. We focused on UCA1 because a) qRT-PCR results validating the gene array data showed that UCA1 expression was increased more than 100-fold in DU145-IRR cells compared with DU145-Parental cells (Supplementary Figure S2G), b) UCA1 is a functional lncRNA involved in several cancer-related biologic processes including tumor growth, invasion and metastasis, and drug resistance [11], and c) UCA1's potential role in conferring radioresistance has not been investigated. Moreover, as it is described in only one publication to date, UCA1 expression level is increased in PCa tumors which may contribute to PCa progression [15]

**Table 1:** List of the top 10 up- and top 10 down-regulated genes in DU145-IRR compared to DU145-Parental cells

Gene Symbol	Mean Fold Change	log2 Fold Change
SEMA3E	25.84	4.69
UCA1	10.58	3.40
IGJ	8.12	2.96
PLAC8	6.10	2.60
FBN1	5.50	2.46
APBB1IP	4.99	2.31
DPYD	4.93	2.28
MATN2	3.45	1.79
NRCAM	3.45	1.78
LEPREL1	3.35	1.75
BCHE	0.05	−4.39
SCEL	0.06	−4.08
NFIB	0.09	−3.43
NRG4	0.20	−2.35
TC2N	0.21	−2.23
HIST1H3G	0.21	−2.52
CDH1	0.22	−2.22
ZNF826P	0.22	−2.20
ZNF320	0.23	−2.12
ZNF888	0.23	−2.14

### UCA1 knockdown reverses radioresistance phenotype

**Figure 2:**
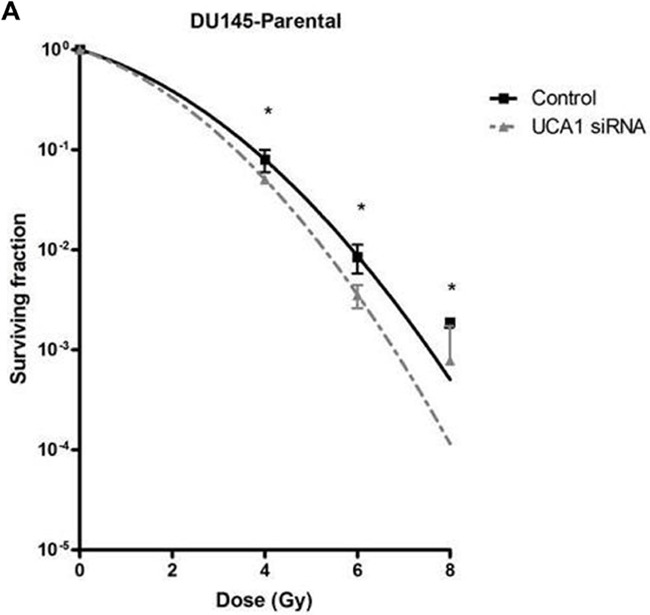

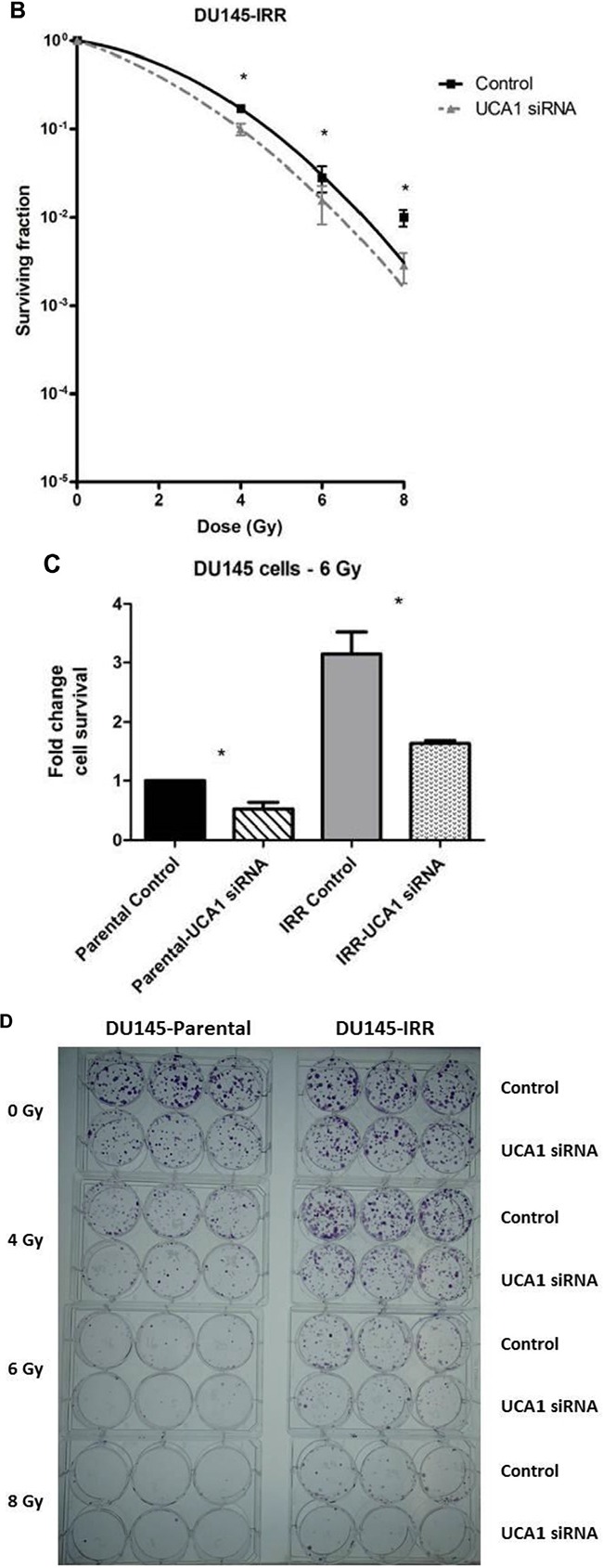

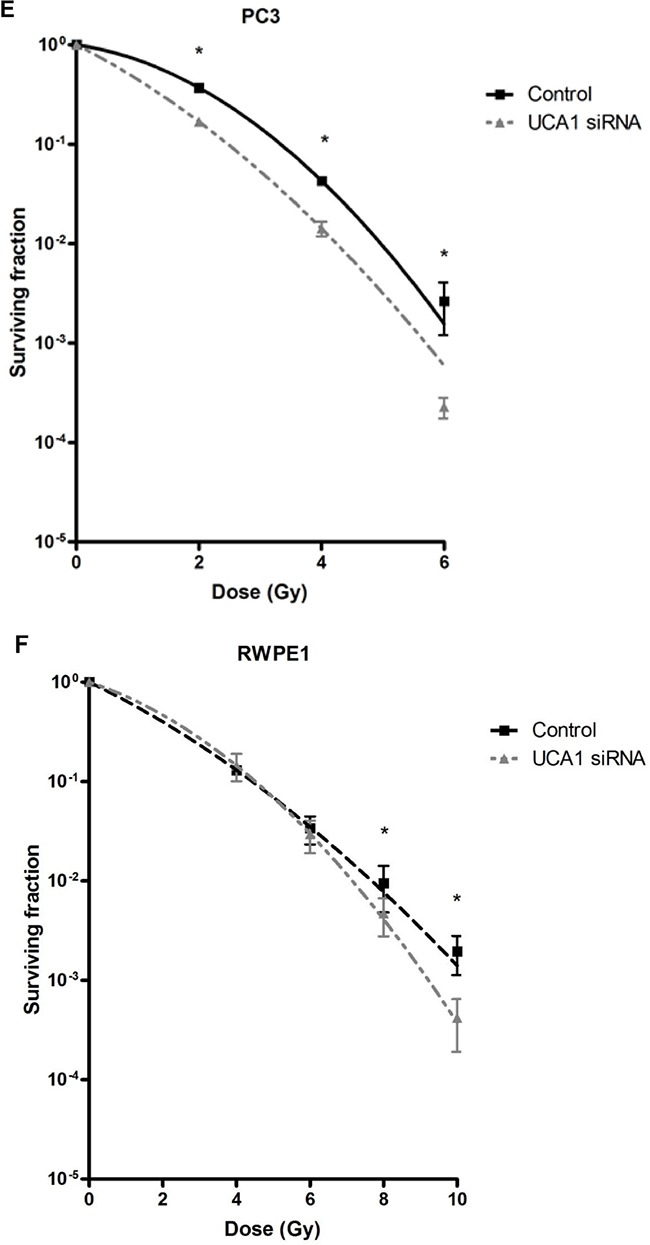
UCA1 knockdown improves radiosensitivity **A**. DU145-Parental, **B**. DU145-IRR, **C**. The relative ratio of surviving DU145-Parental and DU145-IRR cells transfected with UCA1 siRNA following 6Gy of IR compared to corresponding controls. **D**. A representative clonogenic survival assay out of three experiments. Means, SDs, and differences of statistical significance are denoted by * (p < 0.05); n = 3 independent experiments for each experiment. **E**. PC3 and **F**. RWPE1 cells were transiently transfected with control or UCA1 siRNA, radiation clonogenic survival assays were performed, and surviving fraction fitted to the linear-quadratic equation. There were statistically significant differences in cell survival following 4 Gy, 6 Gy, 8 Gy and 10 Gy (RWPE1) of IR for all survival curves (p < 0.05).

We utilized a siRNA knockdown strategy to investigate the potential role of UCA1 in conferring IRR. DU145-Parental and DU145-IRR cells were transiently transfected with a UCA1 siRNA (n272526) or a control siRNA and radiation clonogenic survival assays were performed. Both DU145-Parental-UCA1 siRNA and DU145-IRR-UCA1 siRNA cells displayed significantly decreased radiation survival to an acute exposure to 4 Gy, 6 Gy and 8 Gy of IR compared with control siRNA cells (Figure 2A and 2B; ANOVA and t-test; p values < 0.05). Figure 2C shows the relative ratio of surviving DU145-Parental-UCA1 siRNA and DU145-IRR-UCA1 siRNA cells following 6Gy of IR compared to corresponding controls. A representative experiment out of three clonogenic survival experiments is shown in Figure 2D. Transfection of a UCA1 siRNA was also able to induce radiation sensitivity in an additional PCa cell line (PC3) and a human prostatic epithelial cell line (RWPE1) (Figure 2E and 2F; ANOVA and t-test; p values < 0.05). We performed radiation clonogenic survival assays with two other UCA1 siRNAs (n272528 and n272529) targeting different regions within UCA1 in two different PCa cell lines. These experiments further confirmed that knockdown of UCA1 was able to re-sensitize IRR PCa cells to radiation, in addition to cells not previously rendered resistant to radiation (Supplementary Figure S2A-S2F). Therefore, the rest of the experiments were performed using UCA1 siRNAs (n272526). The knockdown effect of UCA1 siRNAs in DU145 and PC3 cells is shown in Supplementary Figure S2G and S2H, respectively. We also investigated a highly differentially expressed SEMA3E gene, but did not observe any difference in radiosensitisation with a knockdown approach (Supplementary Figure S3A and S3B). The knockdown effect of SEMA3E siRNA in DU145-Parental and DU145-IRR cells is shown in Supplementary Figure S3C and S3D, respectively.

**Figure 3:**
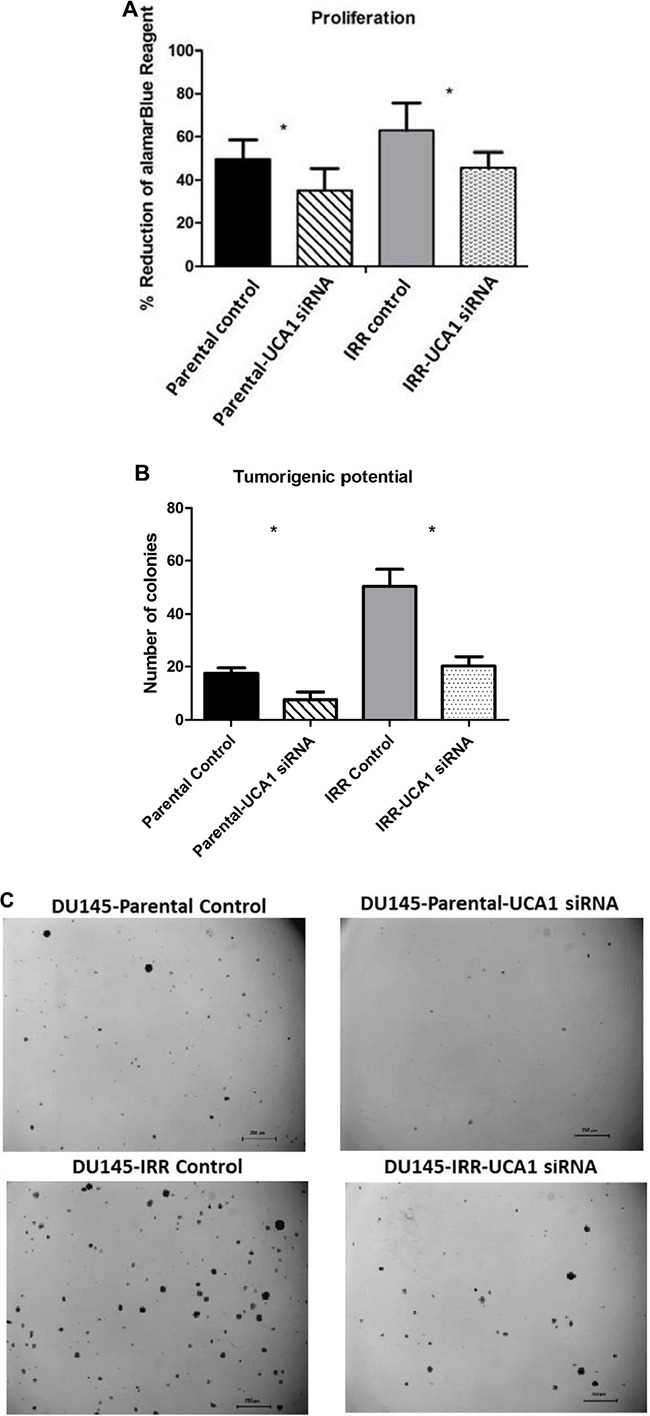

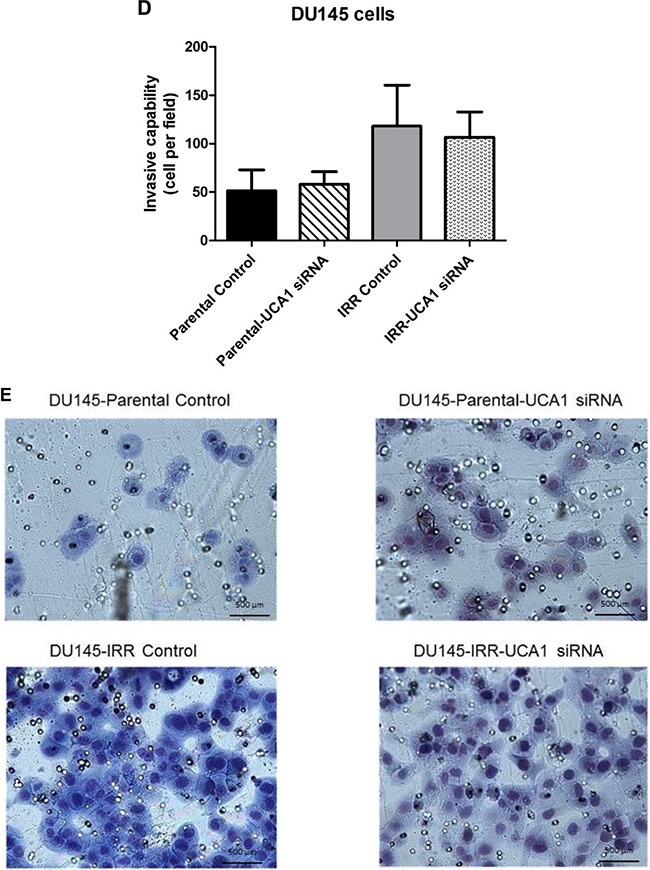
UCA1 knockdown reverses aggressive phenotype **A**. A cell proliferation assay using alamarBlue shows significantly lower level of reduced alamarBlue in DU145-Parental and DU145-IRR cells transfected with UCA1 siRNA (p < 0.05). **B**. Soft agar assay of DU145-Parental and DU145-IRR cells transfected with UCA1 siRNA compared to corresponding controls. **C**. A representative soft agar experiment out of three experiments (scale bar denotes 200μm). **D**. Matrigel transwell invasion assay of DU145-Parental and DU145-IRR cells transfected with UCA1 siRNA compared to corresponding controls. **E**. A representative invasion assay out of three experiments. Means, SDs, and differences of statistical significance are denoted by * (p < 0.05); n = 3 independent experiments for each experiment.

A significant decrease in cellular proliferation was observed in DU145-Parental-UCA1 siRNA and DU145-IRR-UCA1 siRNA cells compared to their corresponding controls (Figure 3A, p values = 0.007 and 0.02, respectively). Similar results were observed in PC3 and RWPE1 cells transfected with UCA1 siRNA (Supplementary Figure S4A and S4B). We then utilized the soft agar assay to evaluate the effect of UCA1 knockdown on tumorigenic potential. As shown in Figure 3B and C, UCA1 knockdown decreased colony formation in both DU145-Parental (mean number of colonies: 7.5 ± 1.4 (UCA1 siRNA) vs. 17.5 ± 1 (control); p = 0.001) and DU145-IRR cells (mean number of colonies: 20.25 ± 1.8 (UCA1 siRNA) vs. 50.5 ± 3.1 (control); p = 0.0002).

To further investigate the mechanism of UCA1 on aggression, we assessed invasiveness following UCA1 knockdown and observed that this did not significantly alter the invasive capacity of DU145-Parental (1.22 ± 0.34 *vs*. 1.0 (control), p = 0.67) or DU145-IRR cells (0.97 ± 0.3 *vs*. 1.0 (control), p = 0.7) (Figure 3D, 3E). Taken together, our results indicate that UCA1 knockdown can largely reverse the aggressive phenotype, modulate radiation sensitivity and inhibit tumorigenic potential.

### UCA1 knockdown effect on cell cycle progression and DNA repair

**Figure 4:**
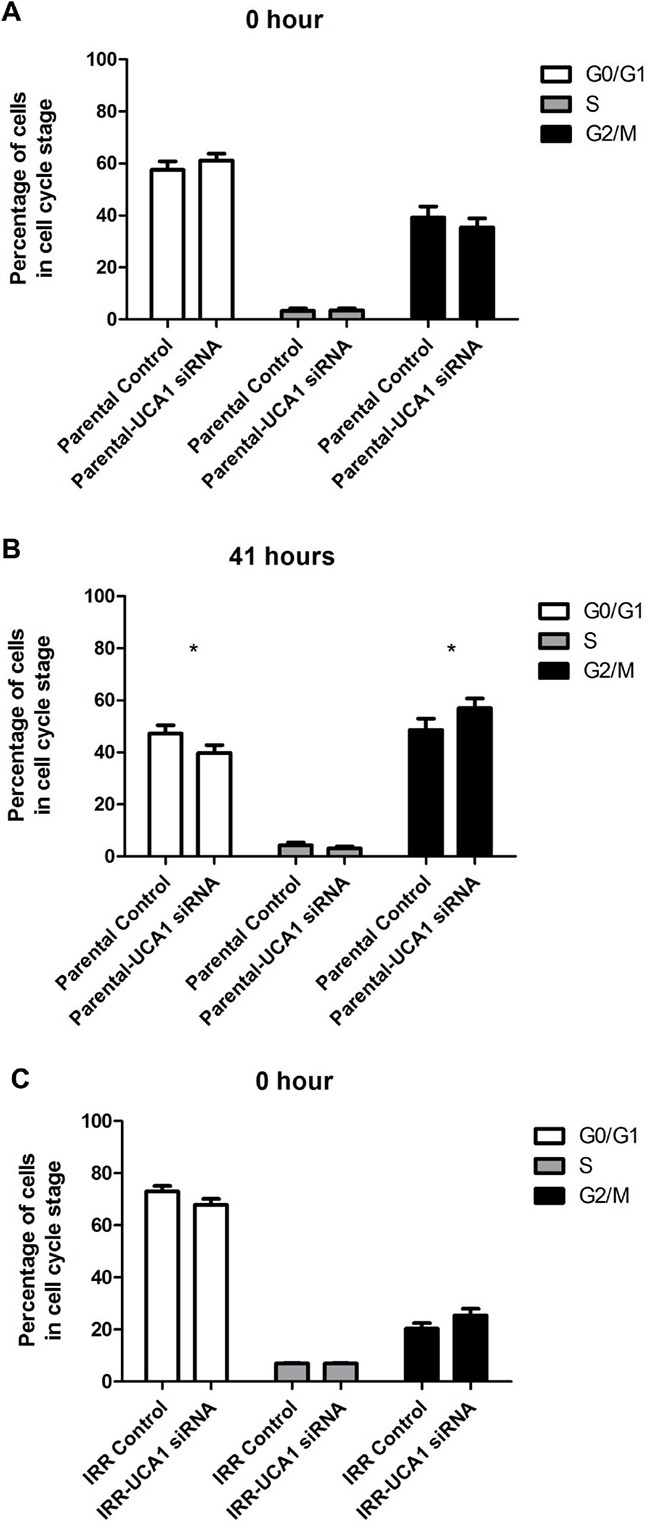

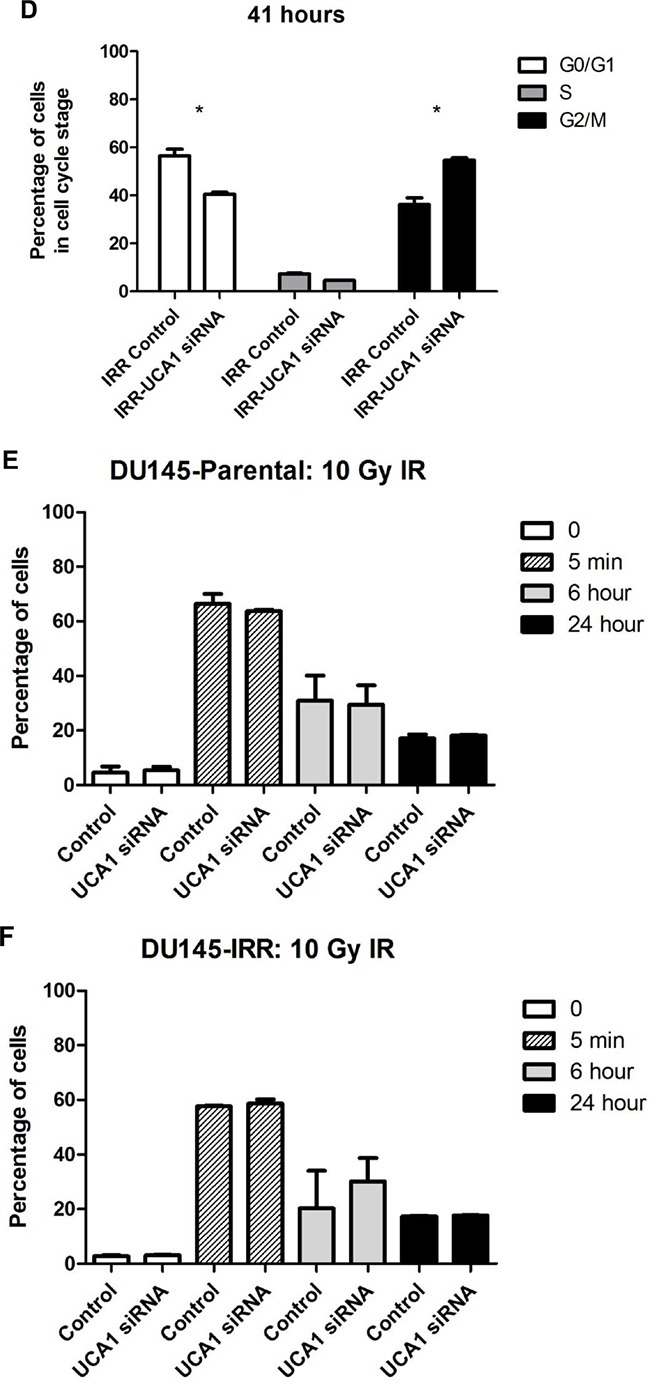
UCA1 depletion disrupts cell cycle progression and induces cell cycle arrest at the G2/M transition Cell-cycle profiles of DU145-Parental **A, B**. and DU145-IRR **C, D**. cells transfected with UCA1 siRNA following a 6 Gy dose of IR at 0 hour or 41 hours, respectively. Staining for gamma-H2AX showed no significant difference in DSB repair kinetics following 10 Gy IR in **(C, D)** cells transfected with UCA1 siRNA following a 6 Gy dose of IR at 0 hour or 41 hours, respectively. Staining for gamma-H2AX showed no significant difference in DSB repair kinetics following 10 Gy IR in **E**. DU145-Parental and **F**. DU145-IRR cells transfected with UCA1 siRNA. Means, SDs, and differences of statistical significance are denoted by * (p < 0.05); n = 3 independent experiments for each experiment.

Flow cytometry analysis was performed to investigate the influence of UCA1 depletion on cell cycle distribution. 41 hours after IR, DU145-Parental-UCA1 siRNA cells showed a significantly decreased proportion in the G0/G1 phase [G0/G1 phase: 39.8 ± 4.1% (UCA1 siRNA) *vs*. 47.2 ± 4.6% (control); p = 0.006], no change in the S phase [S phase: 3.14 ± 0.9% (UCA1 siRNA) *vs*. 4.3 ± 1.5% (control); p = 0.57] and a significantly increased proportion in the G2/M phase [G2/M phase: 57.1 ± 5.1% (UCA1 siRNA) *vs*. 48.6 ± 6.1% (control); p = 0.01] compared to control cells (Figure 4A, 4B). Consistently, we observed that UCA1 knockdown significantly decreased the ratio of cells in the G0/G1 phase [G0/G1 phase: 40.6 ± 1% (UCA1 siRNA) *vs*. 56.5 ± 4% (control); p = 0.005], did not change the ratio of cells in the S phase [S phase: 4.6 ± 0.2% (UCA1 siRNA) *vs*. 7.3 ± 0.4% (control); p = 0.6], but increased the cells in the G2/M phase in DU145-IRR cells [G2/M phase: 54.7 ± 1.3% (UCA1 siRNA) *vs*. 36.2 ± 4% (control); p = 0.009], 41 hours after irradiation exposure (Figure 4C, 4D). Our results suggest that UCA1 can induce cells to accumulate in the G2/M phase, which likely contributes to the radiosensitisation resulting from UCA1 knockdown.

We also performed a flow cytometry assay of γ-H2AX to investigate the effect of UCA1 knockdown on DNA double-stranded break (DSB) resolution. The kinetics of γ-H2AX resolution after IR is related in part to intrinsic radiosensitivity. The rapid disappearance of γ-H2AX, with the passage of time after exposure to IRR, is generally associated with resistance to RT. [16, 17] The percentage of γ-H2AX staining cells was similar for DU145-Parental-UCA siRNA and DU145-Parental control siRNA cells at 5 min, 6 hours and 24 hours following 10 Gy IR (p values = ns; Figure 4E). The percentage of γ-H2AX staining cells was higher at 6 hours following IR in DU145-IRR-UCA1 siRNA cells relative to DU145-IRR control siRNA cells; however this difference did not reach statistical significance (p value = 0.27; Figure 4F). These results indicate that UCA1 knockdown does not significantly alter DNA DSB repair kinetics after IR and thus this mechanism does not contribute to radiosensitization.

### Knockdown of UCA1 results in distinct alterations of phospho-kinase activity

**Figure 5:**
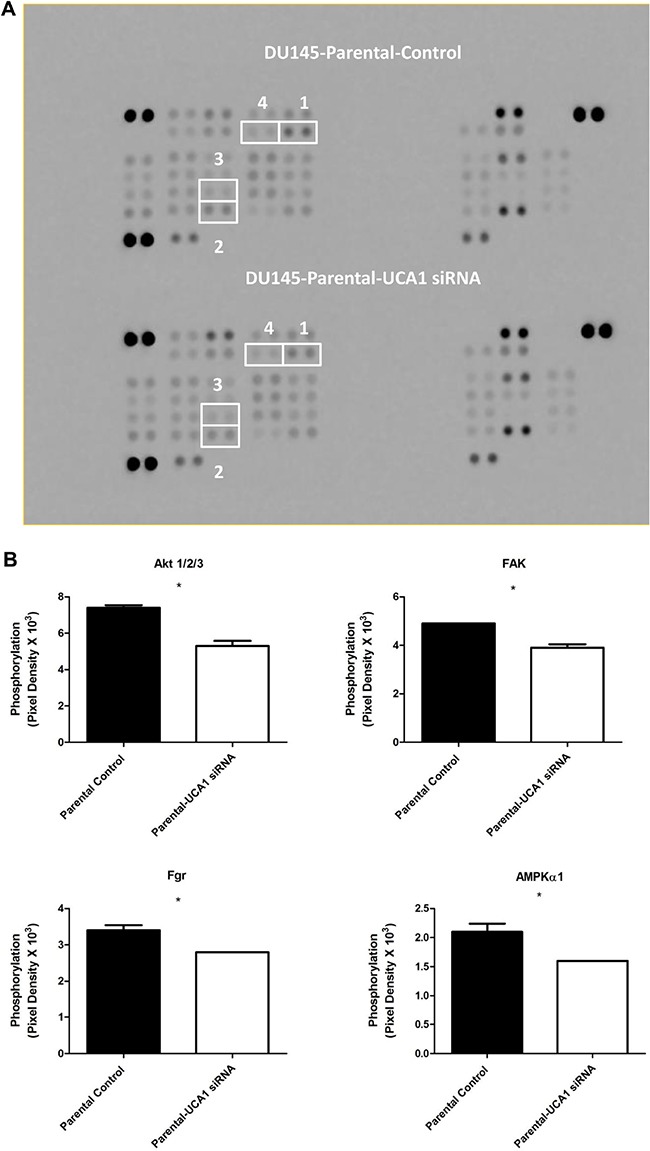

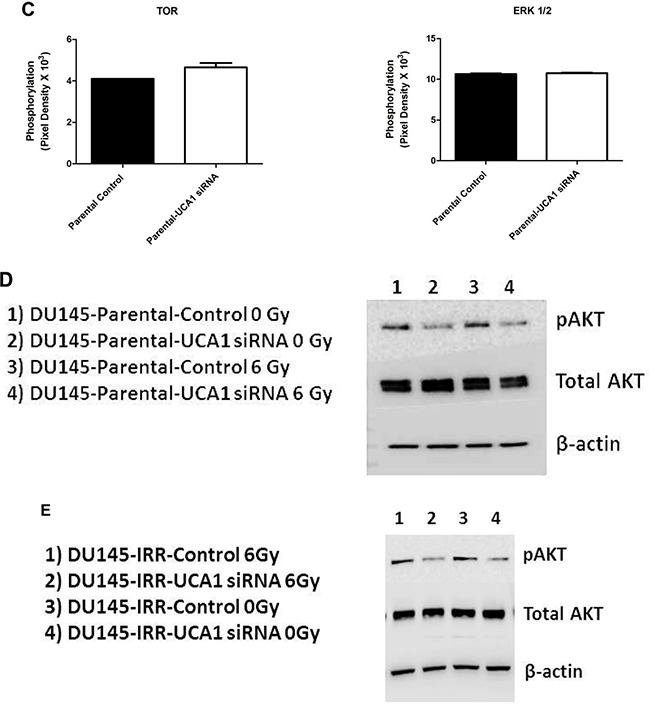
Screening of the phosphorylation status of various protein kinases and Western blotting analysis in DU145-Parental and DU145-IRR cells non-transfected and transfected with UCA1 siRNA **A**. Images of the phospho-kinase membrane array [1. Akt 1/2/3 (S473), 2. FAK (Y397), 3. Fgr (Y412), and 4. AMPKα1 (T183)]. **B**. Corresponding profiles of these analytes showing significantly reduced phosphorylation. C. mTOR (S2448) and ERK (T202/Y204, T185/Y187) phosphorylation were unaffected. **C**. mTOR (S2448) and ERK (T202/Y204, T185/Y187) phosphorylation were unaffected. **D, E**. Representative western blots for phosphorylated AKT (pAKT), total AKT and β-actin (loading control) levels in (D) DU145-Parental and in (E) DU145-IRR cells transiently transfected with UCA1 siRNA (2, 4) or control (1, 3) before and after 6 Gy IR. Means, SDs, and differences of statistical significance are denoted by * (p < 0.05).

The process of radiosensitization is complex and involves alterations in proliferative and pro-survival signaling pathways. Given the complexity and potential cross-talk between these pathways, we utilized a phospho-kinase array to identify changes in key signaling events associated with UCA1 depletion. Protein lysates collected from DU145-Parental-UCA1 siRNA and control siRNA cells were subjected to this array and the relative site-specific phosphorylation of 39 individual kinases involved in cellular proliferation and survival were assessed (Figure 5A). Significantly reduced phospho-specific activation of several kinases was seen in UCA1 knockdown cells compared to control cells. Figure 5B presents these notable changes. These kinases include Akt (PI3K/Akt pathway), FAK (Focal Adhesion Kinase), Fgr kinase, and AMPKα1 (AMP-activated protein kinase); these kinases have been implicated in promoting survival after RT, tumor progression and metastasis processes (*e.g*., migration and cellular adhesion signaling) [2, 18, 19]. Although mTOR is prototypically activated via Akt, we observed an increased trend in mTOR-S2448 phosphorylation in UCA1 knockdown cells rendering a less aggressive phenotype (Figure 5C). It is now appreciated that mTOR can also receive cues from other signaling pathways (*i.e*., in an Akt-independent manner) [20]. Recent data also suggest that the mTOR signaling pathway activation (evidenced by S2448 phosphorylation) is associated with a favorable prognosis in PCa [21, 22]

Although inhibition of ERK signaling can promote cancer radiosensitization, [23] we did not detect altered ERK 1/2 (T202/Y204, T185/Y187) phosphorylation (Figure 5C), and thus this pathway is likely not contributory to UCA1 radiosensitization. Western blotting analyses confirmed the phospho-kinase array findings related to mTOR and ERK phosphorylation (data not shown). We noted reduction in Fgr phosphorylation; however, we did not observe significant changes in phosphorylation of other Src family kinases, including Src, Yes, Fyn, Lck, Hck, and Lyn (Supplementary Figure S5).

The PI3K/Akt pathway is one of the major signal transduction pathways regulating cell proliferation, survival and progression, and it plays a central role in PCa radioresistance. [2, 24] It is frequently activated in cancer cells and promotes cellular proliferation and blocks cell death through activation of different downstream effectors. [25] Accordingly, alteration in phosphorylated-Akt (pAkt) was the most notable change detected on our array, and given its importance in cancer progression and therapy response, we proceeded to validate this result with western blotting using pAkt-specific antibodies that were different from those used in the array. We confirmed that knocking down UCA1 expression in DU145-Parental and DU145-IRR cells significantly reduced the level of pAkt compared to their respective controls before and after 6Gy IR (Figure 5D and 5E).

### Higher UCA1 expression is associated with unfavorable outcome in PCa patients

**Figure 6:**
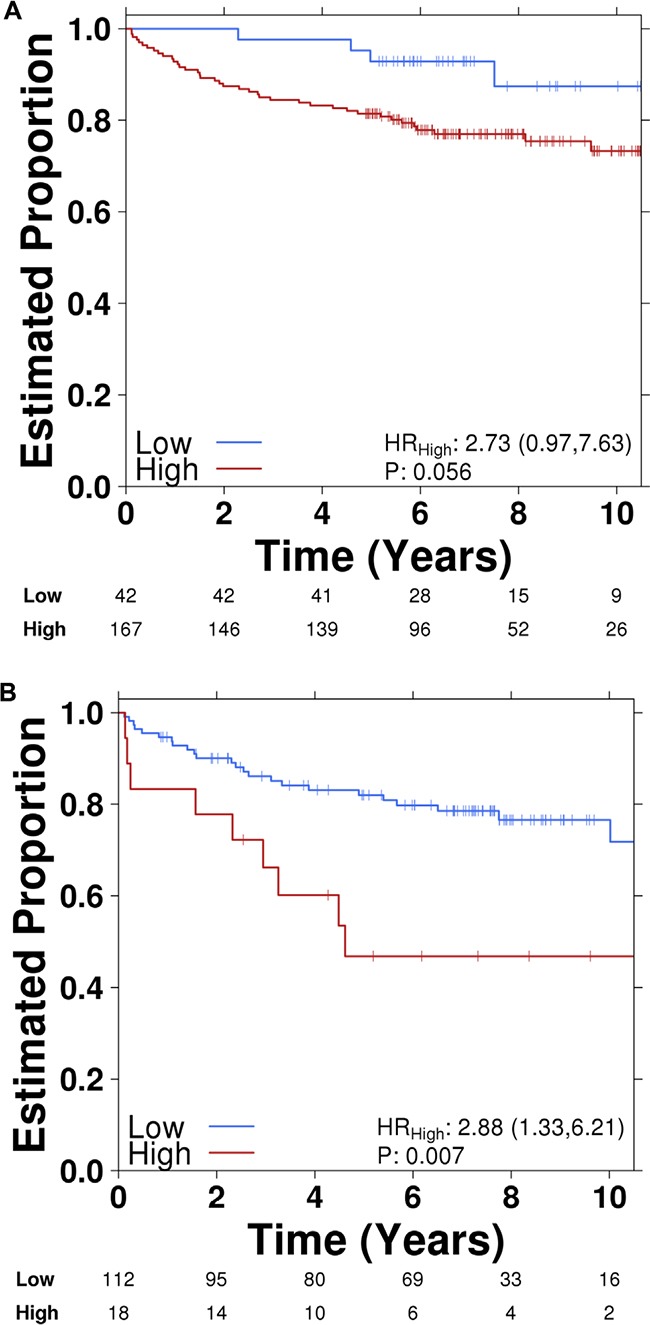

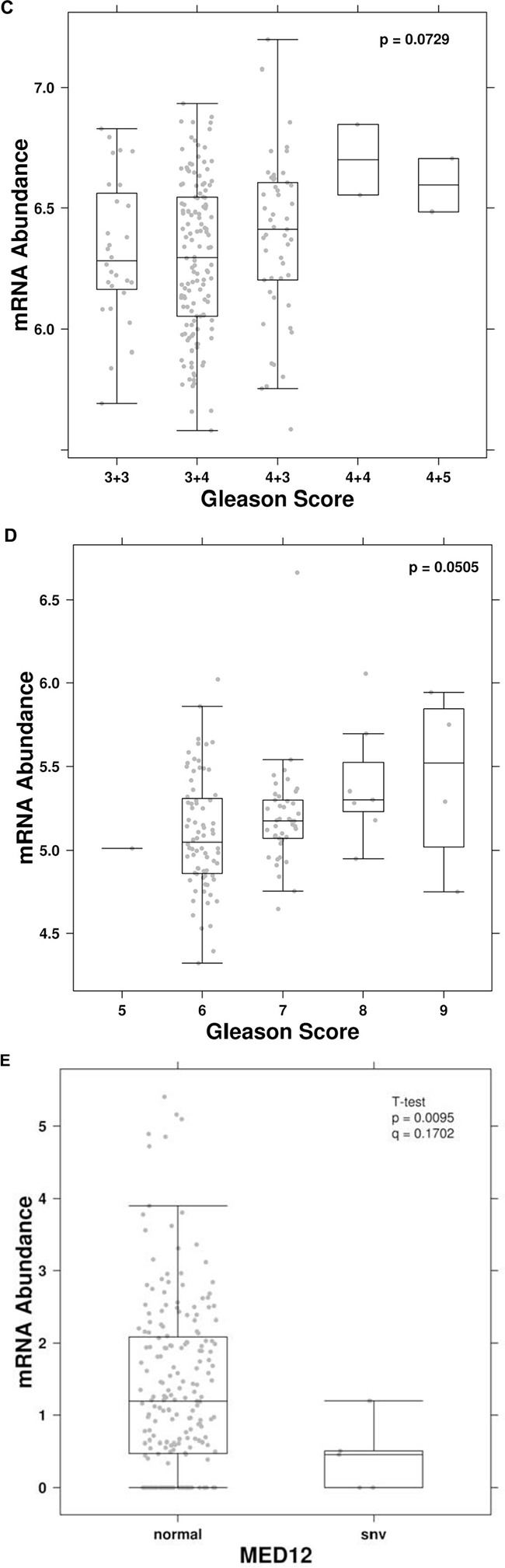
Higher UCA1 expression is correlated with poor PCa outcome Kaplan-Meier plots of biochemical recurrence versus time to recurrence showing the effect of UCA1 expression (high vs. low) in **A**. CPC-GENE and **B**. MSKCC cohorts. Correlation of UCA1 expression with Gleason Score (GS) in. **C**. CPC-GENE and **D**. MSKCC cohorts. **E**. Correlation of UCA1 expression shown as mRNA abundance with MED12 point-mutations.

To determine if UCA1 expression was associated with outcomes in PCa, we first investigated UCA1 expression in the Canadian Prostate Cancer Genome Network (CPC-GENE) data, which includes sequencing data from a cohort of 209 patients with intermediate-risk PCa treated with RT or radical prostatectomy [26]. There was reduced biochemical relapse-free survival in high UCA1 expressor patients compared to low UCA1 expressor patients (81% *vs*. 94%, respectively; cox proportional hazard model, HR = 2.73, p = 0.05; Figure 6A). We next interrogated the publically available MSKCC Prostate Cancer database for mRNA expression data on 130 patients with clinically-staged low- to high-risk PCa who underwent surgical treatment. [27] UCA1 expression was higher in 18 (14%) of 130 patients. Patients with higher UCA1 expression had a significantly reduced disease-free survival at 5 years (5y-DFS) compared to those patients with lower UCA1 expression (5y-DFS = 51% *vs*. 82%, respectively; log-rank test p = 0.007; HR = 2.88; Figure 6B). Furthermore, we observed that higher expression of UCA1 is associated with a trend toward higher Gleason Score (GS) in both CPC-GENE and MSKCC cohorts (ANOVA; p values: 0.07 and 0.05, respectively; Figure 6C and D). However, there was no association between UCA1 expression and pre-treatment PSA (data not shown). We then used TCGA data to determine if UCA1 abundance is associated with recurrent single nucleotide variants (SNV). Interestingly, there was a strong association of UCA1 mRNA with MED12 point-mutations (Figure 6E). MED12 is reported to be recurrently mutated in PCa, [28] and MED12 overexpression is a frequent event in castration-resistant PCa. [29] Our bioinformatics analyses showed that patients with SNV in MED12, which could potentially result in impairment of its function, had lower levels of UCA1 expression (Figure 6E). Taken together, these findings indicate that higher expression of UCA1 is associated with unfavorable outcome in PCa.

## DISCUSSION

Radioresistance remains a significant obstacle in the treatment of many cancers including PCa, limiting treatment success and negatively affecting patient outcomes. LncRNAs are regulators of normal biological processes, neoplastic development and progression, and radiotherapy/chemotherapy resistance [28]. The expression levels of lncRNAs have been associated with carcinogenesis and tumor growth in PCa [29].

UCA1 was originally identified as being overexpressed in bladder cancer and it functions as an oncogenic lncRNA in a variety of different tumor types [10]. We found that UCA1 expression was strikingly high in PCa cells resistant to IR, suggesting its role in therapy resistance. The effect of UCA1 expression in tumor progression, invasion and metastasis is previously described [11]. However, the biological role and underlying mechanism of UCA1 in RT response has remained undefined. We discovered UCA1 as one of the important mediators of radiation response. Using a siRNA knockdown strategy, we further demonstrated that UCA1 knockdown could improve radiosensitivity in both classic PCa cell lines and IRR-PCa cells, and largely reverse the aggressive phenotype observed. To our knowledge, this is the first study investigating the impact of UCA1 on response to radiation in cancer cells.

Molecular mechanisms contributing to therapy resistance in PCa are complex. Recent evidence suggest that PI3K/Akt/phosphatase and tensin homolog (PTEN)/mammalian target of rapamycin (mTOR) signaling pathway, autophagy, epithelial-mesenchymal transition (EMT) and cancer stem cells (CSCs) play important roles in PCa tumorigenesis and may collectively contribute to radioresistance [2]. Activation of the PI3K/Akt pathway confers cancer resistance to radiation [2], and inhibition of this pathway can re-sensitize PCa cells to IR [30]. Previous data indicates that the PI3K/Akt/mTOR pathway is the most activated pathway associated with radioresistance in three PCa-IRR cell lines (DU145-IRR, PC3-IRR, and LNCaP-IRR) developed by irradiating these with 2 Gy per day for only five consecutive days [31, 32]. We believe that our DU145-IRR cells, which have survived a significantly higher total dose of RT delivered over several weeks, are representative of a radioresistant phenotype that best mimics the clinical scenario. In a search for the mechanisms responsible for this radiosensitization, we found that UCA1 knockdown reduced Akt activation.

Our results suggest that UCA1 knockdown reduces Akt activation and consequently improves sensitivity to radiation. The process of radiation resistance involves many molecular pathways. The expression and dysregulation of lncRNAs are highly cancer type specific. [7] UCA1 may regulate therapy response in PCa tumors through its effects on different target genes. The selection of targets may also vary depending on physiological contexts or distinct tumor microenvironment.

The effect of UCA1 on radiation response appears to be partly related to its role in cell cycle regulation. In bladder carcinoma cells, UCA1 regulates cell cycle progression through CREB and via PI3K-AKT-dependent signalling pathways. [33] Our findings similarly suggest that UCA1 can modulate radiosensitivity of PCa cells by impairing cell cycle progression, potentially through downregulation of the PI3K/Akt pathway. Future experiments employing RNAseq to capture the transcriptomic landscape regulated by UCA1 may contribute to additional mechanistic findings.

UCA1 knockdown reversed the aggressive phenotype in DU145 and PC3 PCa cells. The findings that UCA1 knockdown decreased proliferation and tumorigenesis without affecting cellular invasion are consistent with previous studies indicating that proliferation and invasion are two distinct processes under the control of different signaling pathways [34–36]. For example, it has been demonstrated in glioma that invasion, migration and branching morphogenesis are exclusive characteristics of highly invasive cells while highly proliferative cells are highly tumorigenic and display anchorage-independent growth in soft agar [34].

LncRNAs can be used as diagnostic and prognostic biomarkers for PCa [9, 37]. We showed that UCA1 over-expression was associated with an unfavorable outcome in two separate cohorts of PCa patients including a large number of patients with long follow-up data. These observations were in agreement with a recent study by Na *et al*. on a small cohort of 40 patients treated with surgery reporting that patients with high UCA1 levels had poorer prognosis (lower 5-years overall survival) [14]. These findings support further investigations into UCA1 as a prognostic biomarker in other PCa patient cohorts. UCA1 has been shown to serve as a potential diagnostic biomarker for bladder, lung and gastric malignancies. [11] In our laboratory, we have found that UCA1 expression can be detected and quantitated from PCa patient post-digital rectal examination (DRE) urine samples using qRT-PCR and higher UCA1 expression is associated with higher GS (data not shown). The potential of lncRNAs as diagnostic biomarker approach is beautifully demonstrated by the PCA3 test, which is a validated test based upon the detection of the lncRNA PCA3 [38]. We believe that UCA1 may represent a potential biomarker for PCa diagnosis.

In conclusion, UCA1 may regulate PCa cell proliferation, tumorigenesis, cell cycle progression and radiosensitivity through its effects on different target genes. These target genes could differ among specific tissues and cell types. Although the precise role of UCA1 in PCa development remains unclear; the results from the present study support UCA1 as a novel lncRNA that influences radiation response, and represents a potential biomarker for PCa diagnosis and help guide therapy decisions for patients in the future.

## MATERIALS AND METHODS

### Cell lines and cell culture

Human prostate adenocarcinoma cell lines (DU145 and PC3) and the human histologically normal prostate cell line (RWPE1) were purchased from American Type Culture Collection (ATCC; VA, USA). Early passage DU145 and PC3 cell lines were cultured in Dulbecco's modified Eagle medium (DMEM) containing 4.5 g/L glucose (Invitrogen, Canada) supplemented with 10% fetal bovine serum (FBS) (Invitrogen, Ontario, Canada) and penicillin (100 U/mL) – streptomycin (100 μg/mL) (Invitrogen, Ontario, Canada) (hereafter referred to as 10% DMEM), while RWPE1 cells were cultured in Keratinocyte Serum Free Medium supplemented with bovine pituitary extract and human recombinant epidermal growth factor (Invitrogen, Canada), and maintained in a humidified 37°C incubator with 5% CO_2_ Cell lines were passaged when they reached approximately 80% confluency and were regularly tested with MycoAlert (Lonza, Canada) to ensure the absence of mycoplasma contamination.

### Generation of irradiation-resistant prostate cancer cells

The DU145-IRR PCa cells were generated by treatment with mock irradiation (*i.e*., parental cells) or ionizing radiation (*i.e*., IRR) delivered using 110 kV X-rays from a Faxitron 43855F (Faxitron Bioptics LLC, USA) as a daily 2 Gy dose administered 5 days per week, followed by a 7- to 10-day recovery, with this process repeated for a total of 59 treatments (to simulate the clinical scenario of conventional fractionation RT). Radiation resistance was assayed by clonogenic survival, and we confirmed that the cells maintained this phenotype in culture for at least 4 months.

### Transfection of UCA1 siRNA

A total of 3 × 10^5^ cells were seeded into 6-well plates, then 16 hours later, *Silencer* Select Negative Control siRNA (ThermoFisher; catalog # 4390843) or UCA1 siRNA (ThermoFisher; catalog # n272526 was used to perform all experiments in the manuscript, n272528 and n272529 were used to confirm findings of clonogenic survival assays: 3 separate siRNAs targeting different regions within UCA1) were transiently transfected into cells using Lipofectamine 2000 (Invitrogen, Canada) as per manufacturer's recommendations, and 24 hours later, the assays were performed on the transfected cells.

### Clonogenic survival assay

Cells were seeded at 250, 500, 2,000, and 4,000 cells per well onto a six-well plate in 10% DMEM in triplicate and mock irradiated (0 Gy) or irradiated with 2, 4, 6, 8, 10 Gy dose of IR, respectively. Then, cells were placed in a humidified CO_2_ incubator at 37°C to allow colonies to form. Colonies were stained with crystal violet staining solution [0.5% crystal violet (Sigma-Aldrich, USA), 25% methanol] and counted. Survival was expressed as the relative plating efficiencies of the treated cells compared with that of the control cells. The experiments were performed 3 separate times. Radiation dose-response curves were created by fitting the data to the linear quadratic equation S = e^−αD−βD2^ using GraphPad Prism 5.0 (GraphPad Software Inc., USA), where S is the surviving fraction, α and β are inactivation constants, and D is the dose in Gy. Statistical analysis was done using ANOVA and T-test. The p-values less than 0.05 were considered as significant.

### Cellular proliferation

24 hours after transient transfection of cells with UCA1 siRNA or control siRNA, cells were seeded in triplicate (0.5 × 10^5^ cells/well for mock IR, and 1.0 × 10^5^ for 6 Gy IR) in 10% DMEM in 6-well plates and mock irradiated or irradiated with a 6 Gy dose of IR. Four days later, cells were trypsinized and total viable cell number determined using the Countess automated cell counter (Life Technologies, Canada); cell numbers were normalized relative to control siRNA cells. Alternatively, the alamarBlue assay (ThermoFisher Scientific, USA) was performed on day 4 according to the manufacturer's instructions, and absorbance measured using the Benchmark Plus multiwell plate reader (Bio-Rad Laboratories Inc., USA).

### Soft agar assay

Cells were resuspended in 10% DMEM, and 0.22% (w/v) Agar-A (Bio Basic Inc., Canada) and plated in triplicate on a base layer of 0.36% Agar-A in 6-well plates (12,000 cells/well), and placed in a humidified CO_2_ incubator at 37°C. Twenty-five days later, colonies were counted.

### Matrigel transwell invasion assay

Cells were serum starved overnight (0.1% DMEM), then 2 × 10^5^ cells were seeded on top of 8 μm transwell inserts (BD Biosciences, Canada) with 0.1% DMEM and precoated with Matrigel (Becton, Dickinson and Company, USA); 10% DMEM was used as a chemoattractant. After 24 hours, cells that had invaded through the Matrigel coated transwell inserts were fixed, stained by Kwik-Diff Stain (Thermo Fisher Scientific, Canada) and number of invading cells counted under ×10 using a Leica DM LB2 microscope (Leica Microsystems, Canada).

### Cell cycle analysis

Cells were mock irradiated or irradiated with a 6 Gy dose of IR, then 41 hours later, cells were trypsinized, washed in PBS, and fixed in ice-cold 80% ethanol in Hank's Buffered Salt Solution (HBSS; 137 mM NaCl, 5.4 mM KCl, 0.25 mM Na_2_HPO_4_, 0.44 mM KH_2_PO_4_, 1.3 mM CaCl_2_, 1.0 mM MgSO_4_, 4.2 mM NaHCO_3_) for 30 minutes on ice. Fixed cells were collected by centrifugation, washed twice with PBS, and resuspended in 50 μg/mL propidium iodide (Sigma-Aldrich, USA) with 0.6% NP-40 (Thermo Fisher Scientific) and 0.1 mg/mL RNAse A in HBSS for 30 minutes at room temperature in the dark. Cells were then collected by centrifugation, resuspended in PBS, and 20,000 events captured on a FACSCalibur flow cytometer (BD Biosciences, USA) and cell-cycle profile analyzed using FlowJo 10.0.4 (FlowJo LLC, USA).

### Gamma-H2AX detection

Time course DU145-Parental control and DU145-Parental-UCA1 siRNA samples were fixed in 70% ethanol on ice for 30 min. After washing with PBS, cell pellets were blocked in 0.1%(v/v) Triton X-100 and 1% (v/v) goat serum at room temperature for 1 hour, and then incubated with anti-phosphohistone H2AX (ser-139) antibody (EMD Millipore, MA, USA) overnight at 4°C. Following three washes with PBS, cells were incubated with Alexafluor 488 labelled goat-anti-mouse secondary antibody (Invitrogen, Ontario, Canada) at room temperature in the dark for 1 hour. Cells were washed three times with PBS and nuclei counterstained with 0.5 g/mL DAPI (Invitrogen, Ontario, Canada) in PBS for 10 min in the dark at room temperature, followed by three washes with PBS. Samples were analyzed using flow cytometer FACSCalibur DXP (BD Biosciences).

### Gene array expression

Total RNA was isolated from DU145-Parental control and DU145-IRR stable cell lines using RNeasy Mini Kit (Qiagen) as per manufacturer's instructions, and gene expression profiling performed by The Centre for Applied Genomics (The Hospital for Sick Children, Toronto, Ontario, Canada) using an Affymetrix GeneChip Human Gene 2.0 ST array (Affymetrix). Transcriptomic data were normalized using the default parameters in Affymetrix Expression Console Software (V.1.2).

### Phospho-kinase antibody array

DU145-Parental control siRNA and DU145-Parental-UCA1 siRNA transfected cells were analyzed using the Human Phospho-Kinase Array (R&D Systems, USA: Catalog # ARY003B). This array specifically detects the relative phosphorylation levels of 39 individual proteins involved in cellular proliferation and survival. Cell lysates were incubated with the membrane. A cocktail of biotinylated detection antibodies, streptavidin-horseradish peroxidase and chemiluminescent detection reagents were used to detect the phosphorylated protein. The relative expression of phosphorylated proteins (performed in duplicates) was determined by quantification of scanned X-ray film using ImageJ (version 1.45), based upon pixel density.

### Western blotting

Cells were lysed in ice-cold radioimmunoassay precipitation assay lysis buffer (50 mM Tris pH 7.5, 150 mM NaCl, 2 mM EDTA pH 8.0, 0.5% (v/v) Triton X-100, and Complete protease inhibitor cocktail (Roche, USA)). Cell debris and insoluble material were removed by centrifugation at 12,000 × g at 4°C for 20 minutes. Following protein quantitation using the Bradford protein assay (Bio-Rad, USA), 25 μg of lysate was loaded per lane and proteins resolved by 4% to 20% gradient SDS-PAGE gel, wet-transferred to polyvinylidene fluoride membranes (EMD Millipore, USA), and the membranes were incubated in 5% nonfat dry milk in Tris-buffered saline Tween-20 (TBST; 10 mM Tris-Base, 150 mM NaCl, 0.05% Tween-20; pH 7.4) for 1 hour at room temperature to block nonspecific antibody binding, followed by incubation with primary antibody in 5% milk in TBST overnight at 4°C with gentle agitation. The membranes were washed 3 times for 10 minutes each in TBST, and then incubated in TBST at room temperature for 1 hour, followed by three 10-minute washes with TBST. Protein-antibody binding on the membranes was detected with the use of enhanced chemiluminescence Plus solution (GE Healthcare Life Sciences) followed by exposure of the membranes to X-ray film (FujiFilm, USA). The following antibodies were used: anti-phospho-Akt (Ser473) and anti-total-Akt (Cell Signaling Technology, USA), and anti-β-actin antibody (Santa Cruz Biotechnology, USA).

### Quantitative real-time PCR

Total RNA was extracted using the RNeasy Mini kit (Qiagen, Canada) and cDNA synthesized using SuperScript® VILO™ cDNA Synthesis Kit (ThermoFisher Scientific, Canada; Catalog # 11754250) as per manufacturer's instructions. Gene expression level was quantified through quantitative real-time PCR using the QuantiTect SYBR Green PCR kit (Qiagen, Canada) on the StepOnePlus Real-time PCR system (ThermoFisher Scientific, Canada). Expression levels were calculated using the comparative Ct method via StepOne Software, and relative expression levels normalized to GAPDH. Primer sequences are available in Supplementary Table S1.

### Patient cohort analysis

Using the Canadian Prostate Cancer Genome Network (CPC-GENE) dataset, 209 patients were dichotomized based on UCA1 mRNA abundances and a Cox proportional hazards model was then fit on the patient groups against biochemical recurrence (BCR). The proportional hazard assumption was tested using cox.zph function from survival package. To distinguish “high expression” from “low expression” values, UCA1 expression levels were dichotomized into low (lower 20%) and moderate to high (top 80%). Kaplan-Meier curves were generated based on the dichotomized patient groups. All survival analyses were conducted using survival package (v2.38-3) in the R statistical environment (v3.2.3). Data on 209 prostate adenocarcinoma [PRAD] samples including mRNA abundances, copy number variations, and associated clinical data were downloaded from The Cancer Genome Atlas (TCGA). For each covariate, UCA1 mRNA abundances were divided into the two groups, and a two tailed T-test was used to identify any associations. To determine if recurrent single nucleotide variants (SNV) were associated with UCA1 mRNA abundances, TCGA data were extracted and a T-test was performed using UCA1 abundances against each gene recurrently affected by SNVs. P-values were corrected using FDR. The publically available Memorial Sloan Kettering (MSKCC) Prostate Cancer database (http://cbio.mskcc.org/cancergenomics/prostate/) was interrogated for mRNA expression data on 130 patients with clinically staged localised low- to high-risk PCa who underwent radical prostatectomy [27].

## SUPPLEMENTARY MATERIALS FIGURES AND TABLES




