# Supplementary material for: Long non-coding RNA urothelial carcinoma associated 1 (UCA1) mediates radiation response in prostate cancer
Source: Oncotarget. 2016 Nov 24;8(3):4668–89. doi: 10.18632/oncotarget.13576 (PMC5354863; doi:10.18632/oncotarget.13576)
Supplement: Supplementary file 1 [file oncotarget-08-4668-s001.pdf]

Long non-coding RNA urothelial carcinoma associated 1 (UCA1) mediates radiation response in prostate cancer

SUPPLEMENTARY FIGURES AND TABLE

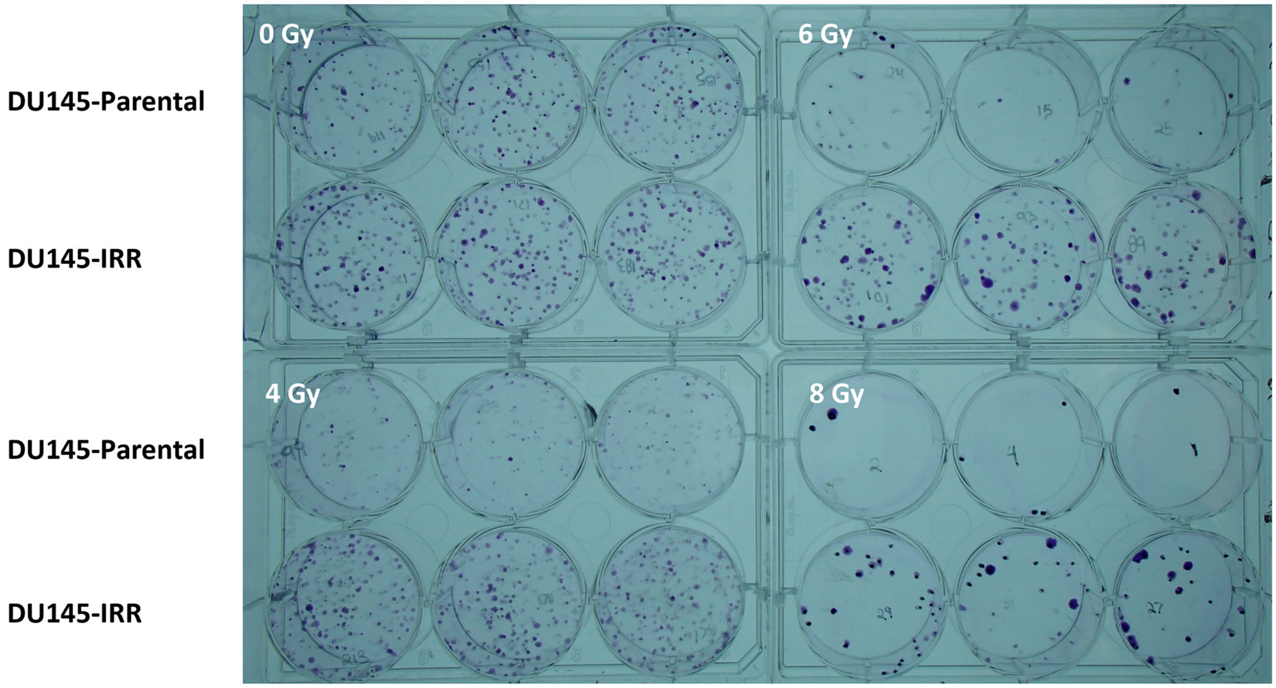

Supplementary Figure S1: A representative clonogenic survival assay out of three experiments in DU145 cells mock irradiated with 0 Gy (DU145-Parental) or irradiated with a total of 2 Gy × 59 daily fractions of IR (DU145-IRR) following 0 Gy, 4 Gy, 6 Gy and 8 Gy of IR.

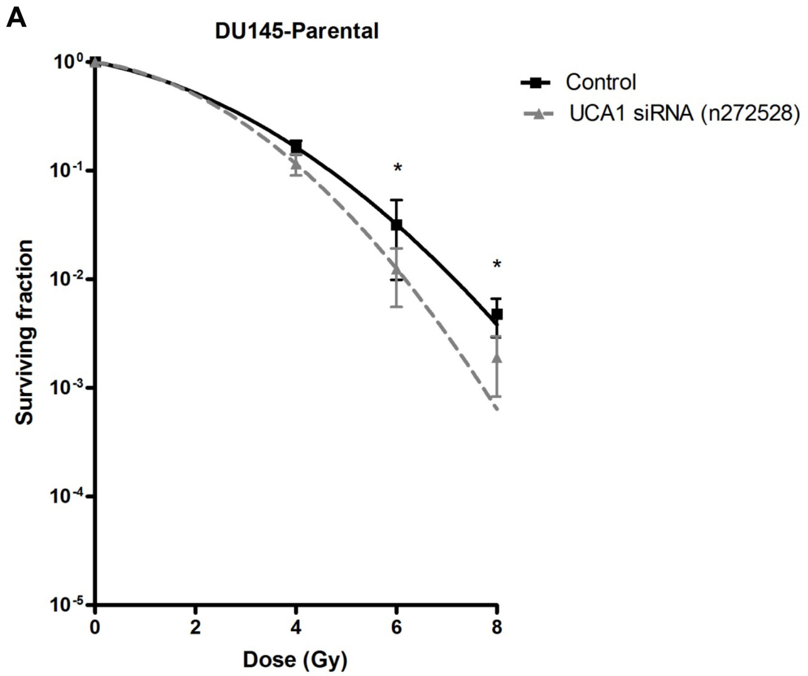

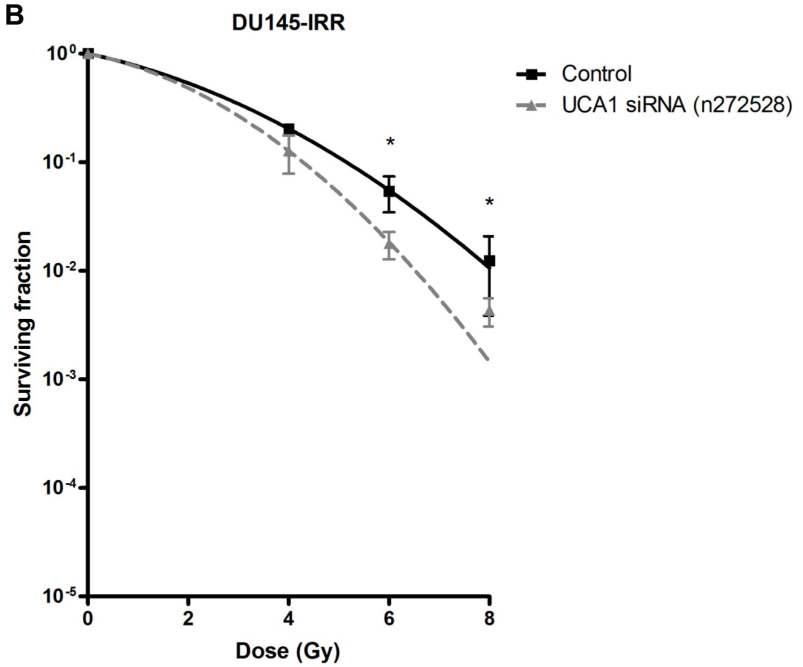

Supplementary Figure S2: The effect of UCA1-knockdown on radiosensitivity was confirmed, using two other different UCA1 siRNAs (n272528 and n272529, respectively) targeting different regions within UCA1, in A, C. DU145-Parental, **B, D.** DU145-IRR, and (*Continued*)

C

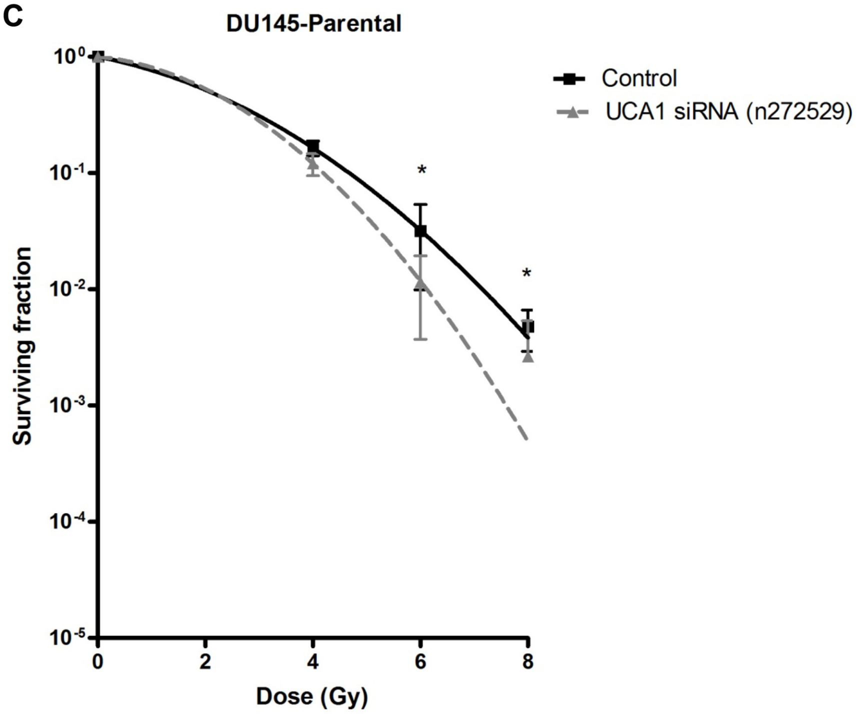

D

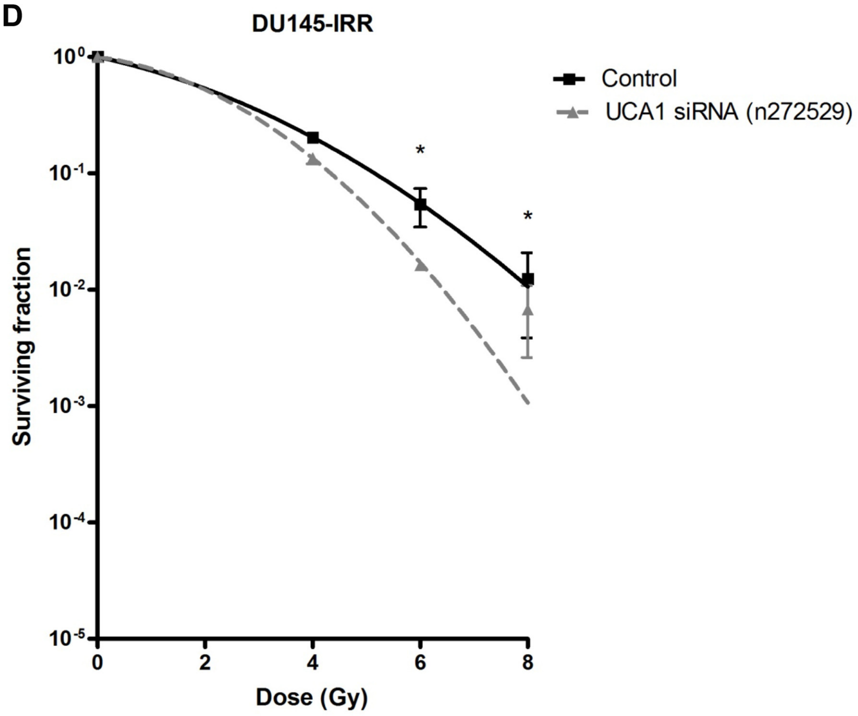

Supplementary Figure S2: The effect of UCA1-knockdown on radiosensitivity was confirmed, using two other different UCA1 siRNAs (n272528 and n272529, respectively) targeting different regions within UCA1, in A, C. DU145-Parental, **B, D.** DU145-IRR, and (*Continued*)

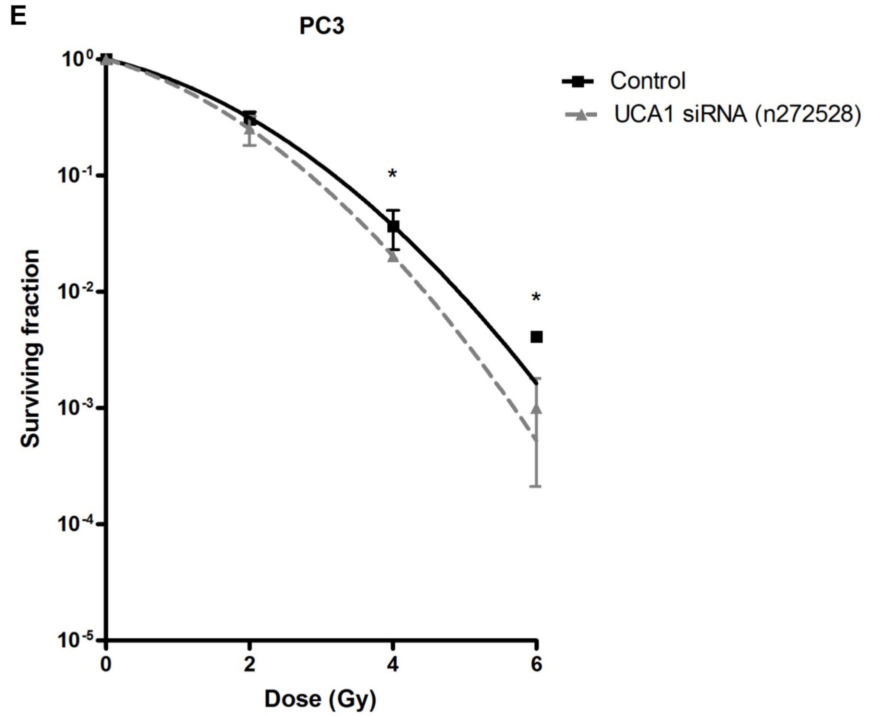

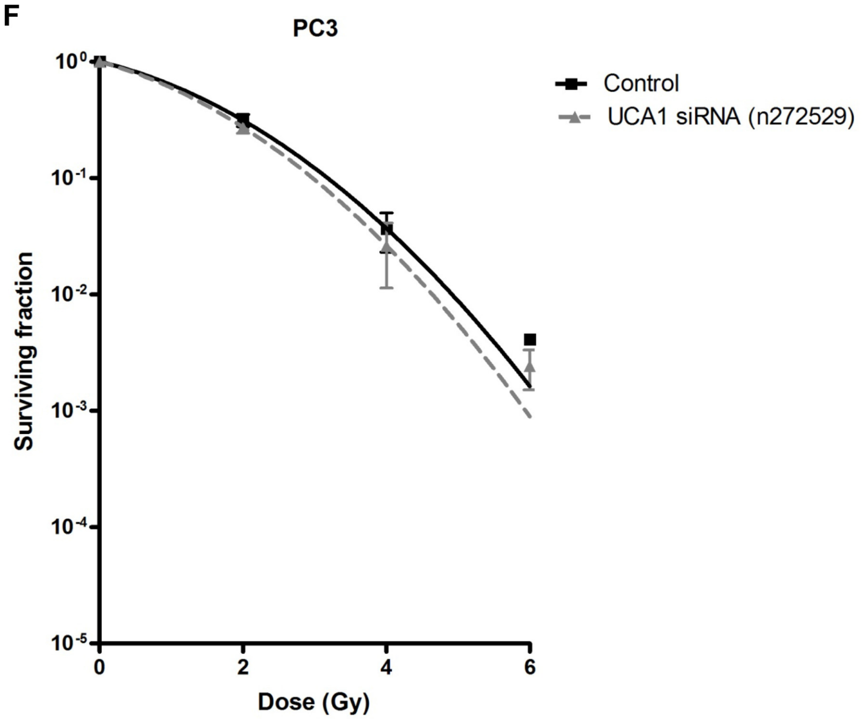

Supplementary Figure S2: The effect of UCA1-knockdown on radiosensitivity was confirmed, using two other different UCA1 siRNAs (n272528 and n272529, respectively) targeting different regions within UCA1, in E, F. PC3. Cells were transiently transfected with control or UCA1 siRNA, radiation clonogenic survival assays were performed, and surviving fraction fitted to the linear-quadratic equation. There were statistically significant differences in cell survival following 4 Gy, 6 Gy and 8 Gy of IR for all survival curves ($p < 0.05$). Means, SDs, and differences of statistical significance are denoted by * ($p < 0.05$); n = 3 independent experiments for each experiment. (*Continued*)

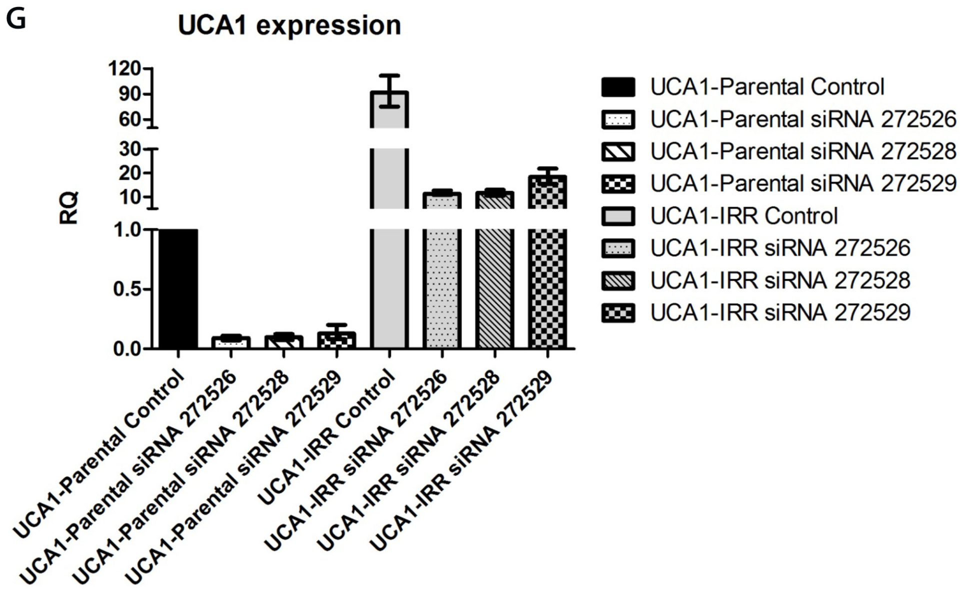

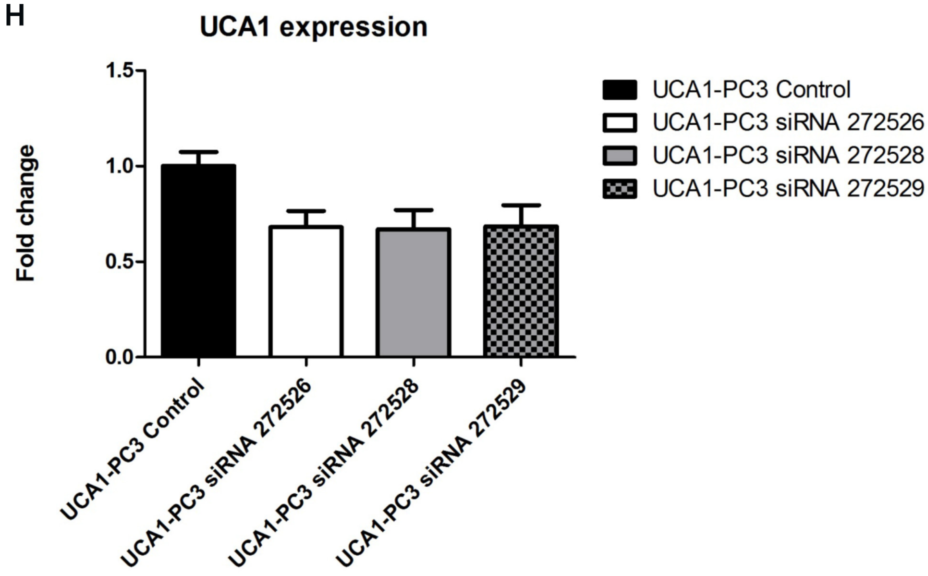

Supplementary Figure S2: The effect of UCA1-knockdown on radiosensitivity was confirmed, using two other different UCA1 siRNAs (n272528 and n272529, respectively) targeting different regions within UCA1, in G. UCA1 expression in DU145-Parental and DU145-IRR and the knockdown effect of UCA1 siRNAs using qRT-PCR. **H.** The knockdown effect of UCA1 siRNAs in PC3 using qRT-PCR.

A

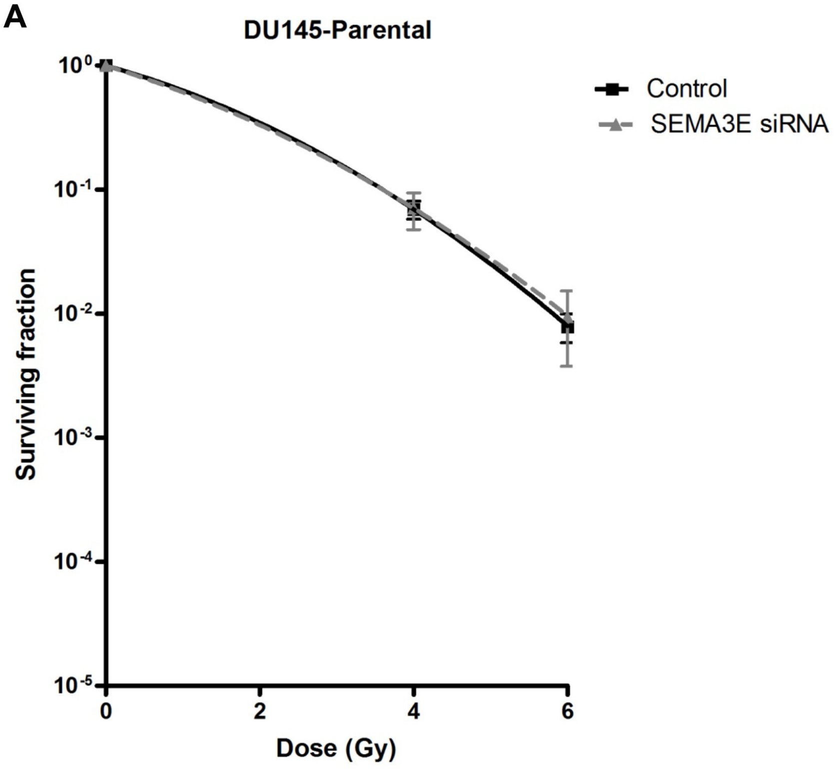

B

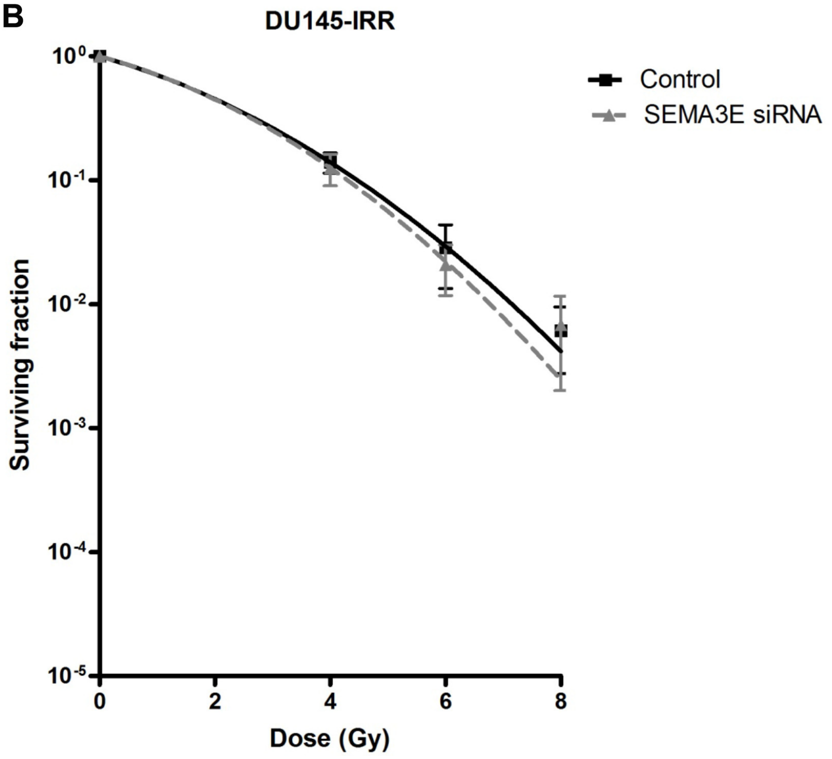

Supplementary Figure S3: SEMA3E-knockdown did not affect radiosensitivity in A. DU145-Parental, **B.** DU145-IRR. Cells were transiently transfected with control or SEMA3E siRNA (Santa Cruz; cat # sc-61520), radiation clonogenic survival assays were performed, and surviving fraction fitted to the linear-quadratic equation. There were no differences in cell survival following 4 Gy, 6 Gy and 8 Gy of IR for all survival curves. The knockdown effect of SEMA3E siRNA in (*Continued*)

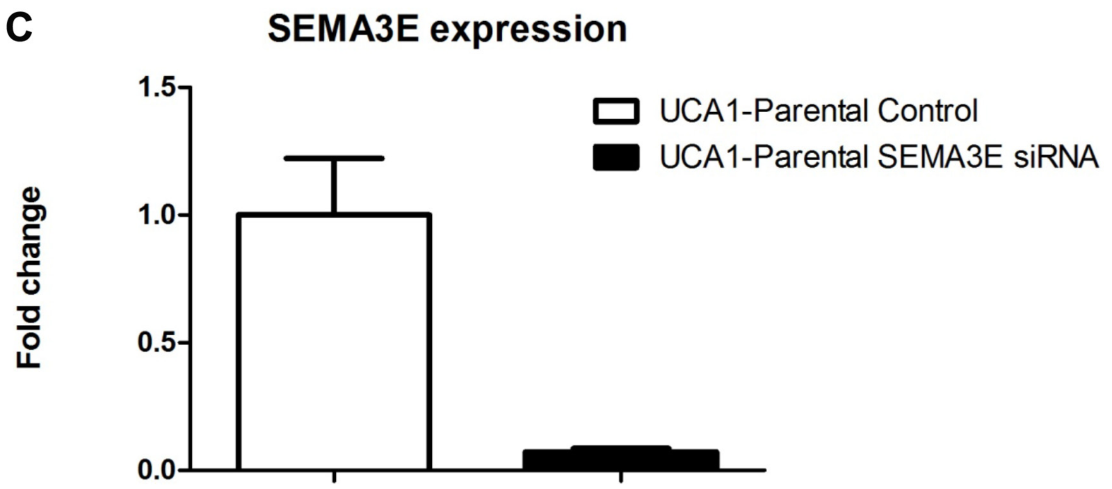

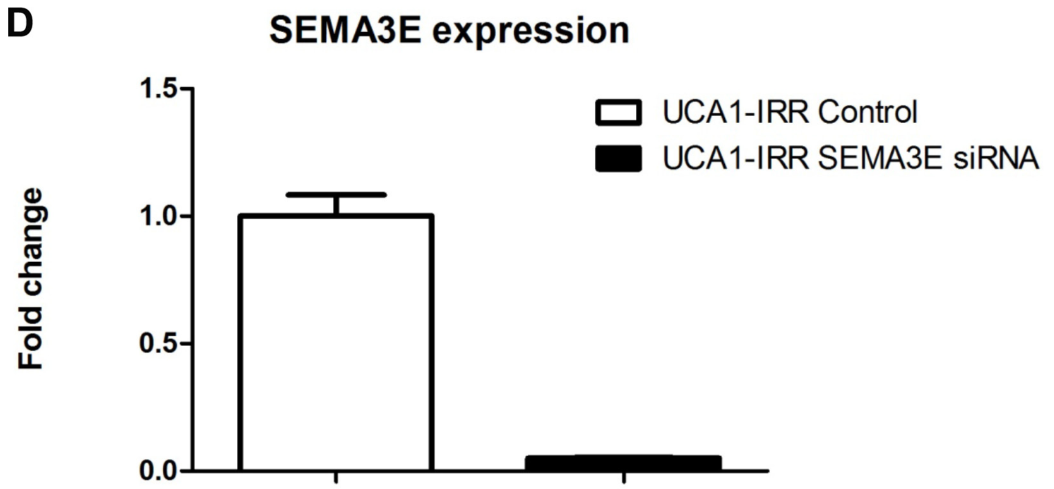

Supplementary Figure S3: SEMA3E-knockdown did not affect radiosensitivity in C. DU145-Parental and **D.** DU145-IRR cells using qRT-PCR. Means and SDs are shown; n = 3 independent experiments for each experiment.

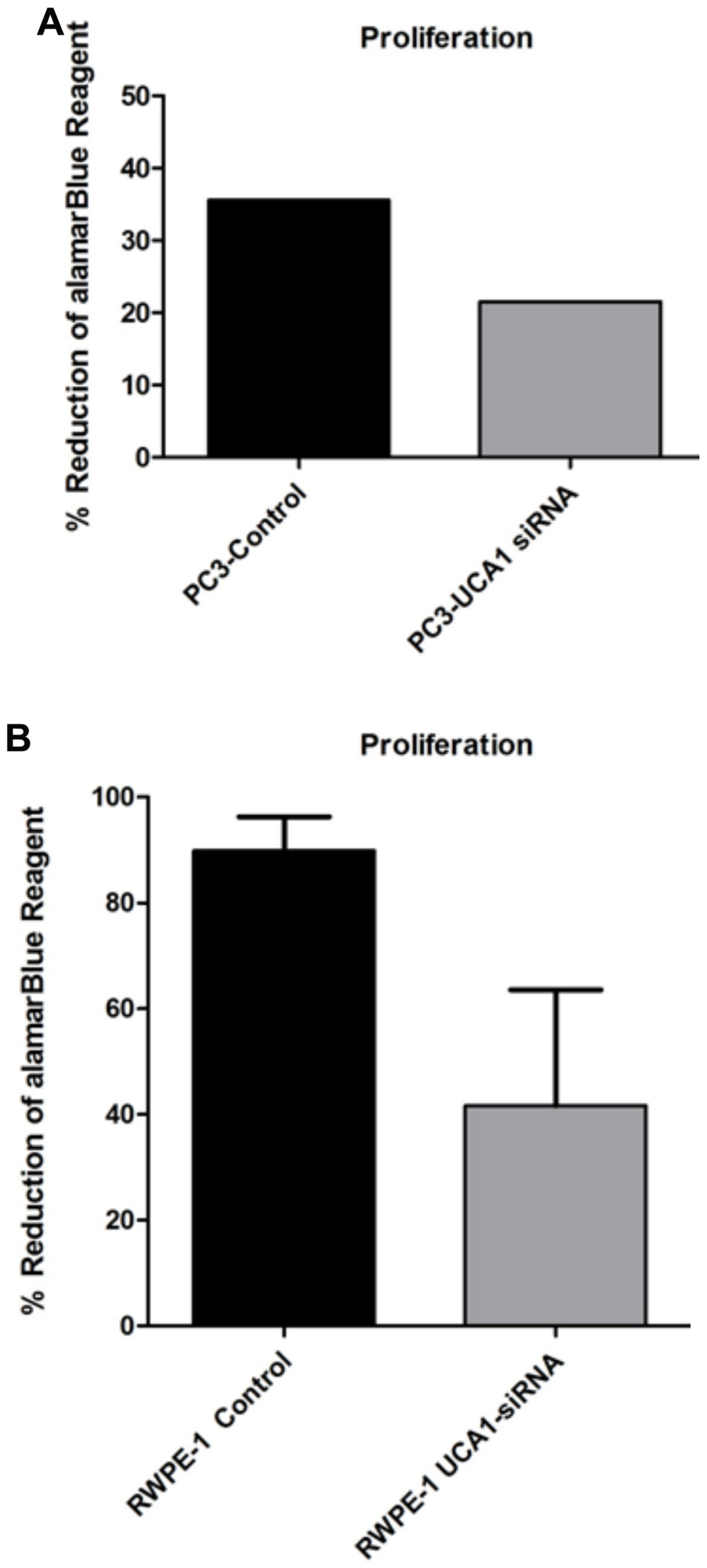

Supplementary Figure S4: A cell proliferation assay using the non-toxic dye alamarBlue shows the lower level of reduced alamarBlue in A. PC3 and **B.** RWPE1 cells transfected with UCA1 siRNA compared to controls.

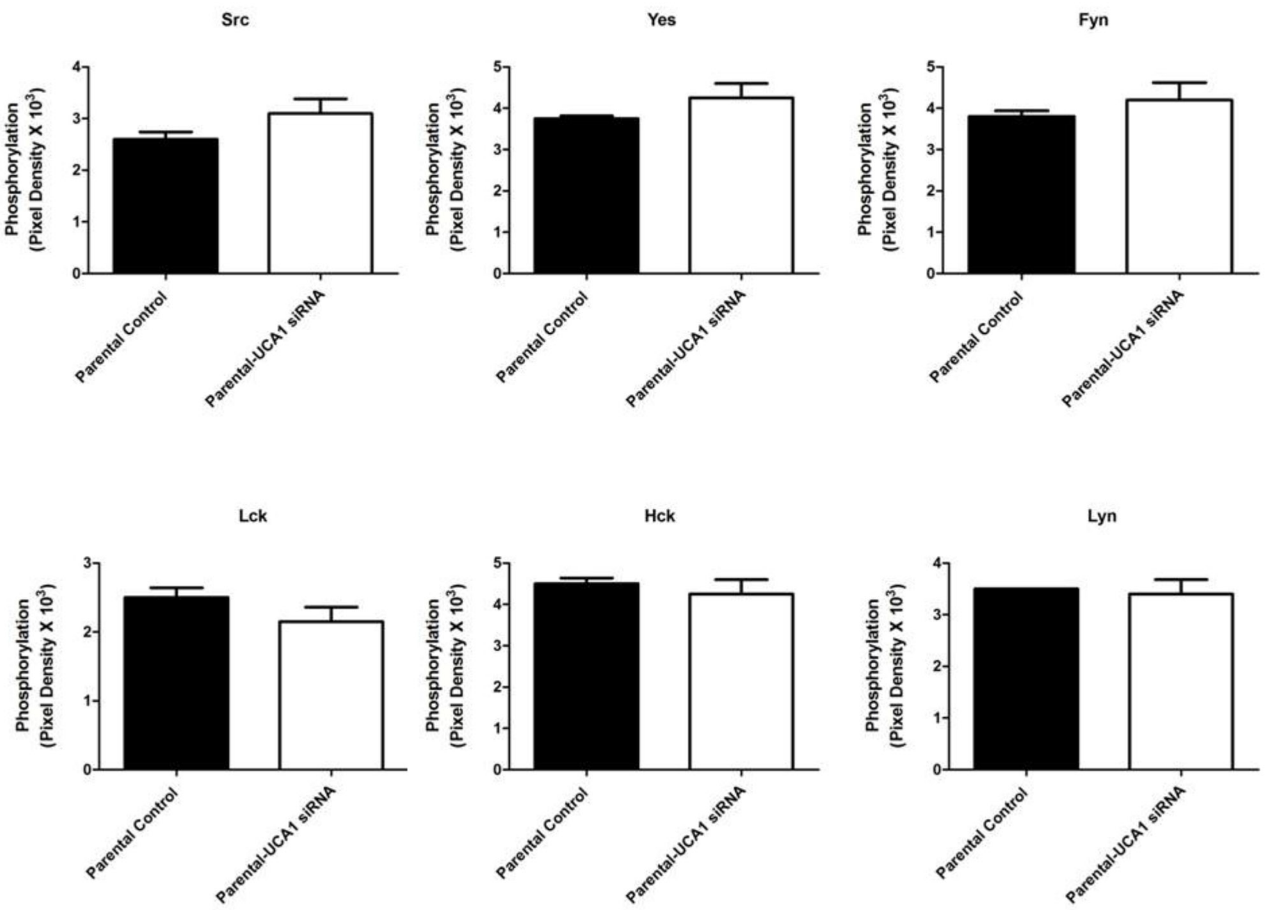

Supplementary Figure S5: Phosphorylation profiles of Src family kinases, including Src, Yes, Fyn, Lck, Hck, and Lyn.

Supplementary Table S1: Primer sequences.

See Supplementary File 1
